# Circular RNA hsa_circ_0062682 Binds to YBX1 and Promotes Oncogenesis in Hepatocellular Carcinoma

**DOI:** 10.3390/cancers14184524

**Published:** 2022-09-19

**Authors:** Rok Razpotnik, Robert Vidmar, Marko Fonović, Damjana Rozman, Tadeja Režen

**Affiliations:** 1 Centre for Functional Genomics and Bio-Chips, Institute of Biochemistry and Molecular Genetics, Faculty of Medicine, University of Ljubljana, 1000 Ljubljana, Slovenia; 2 Department of Biochemistry and Molecular and Structural Biology, Jožef Stefan Institute, 1000 Ljubljana, Slovenia

**Keywords:** circular RNA (circRNA), hepatocellular carcinoma (HCC), YBX1

## Abstract

**Simple Summary:**

Circular RNA (circRNA) have a role in carcinogenesis in different cancers, also in hepatocellular carcinoma (HCC). The transcriptome analyses of HCC tumours identified an upregulated circRNA hsa_circ_0062682. We show that this circRNA affects several aspects of oncogenesis, which are cell proliferation, migration, and invasion. Using transcriptome analyses we identified modulated signalling pathways and transcription factors, confirming the observed phenotype in cells. We identified Y-box-binding protein 1 (YBX1), a known oncogene and RNA-binding protein, as a binding partner, which was in line with transcriptome analyses. We also identified a cell-specific response to sorafenib after circRNA modulation, which is in line with a heterogeneous molecular pathology of HCC subtypes.

**Abstract:**

Circular RNAs (circRNAs) have been shown to play an important role in the pathogenesis of hepatocellular carcinoma (HCC). By implementing available transcriptomic analyses of HCC patients, we identified an upregulated circRNA hsa_circ_0062682. Stable perturbations of hsa_circ_0062682 in Huh-7 and SNU-449 cell lines influenced colony formation, migration, cell proliferation, sorafenib sensitivity, and additionally induced morphological changes in cell lines, indicating an important role of hsa_circ_0062682 in oncogenesis. Pathway enrichment analysis and gene set enrichment analysis of the transcriptome data from hsa_circ_0062682 knockdown explained the observed phenotypes and exposed transcription factors E2F1, Sp1, HIF-1α, and NFκB1 as potential downstream targets. Biotinylated oligonucleotide pulldown combined with proteomic analyses identified protein interaction partners of which YBX1, a known oncogene, was confirmed by RNA immunoprecipitation. Furthermore, we discovered a complex cell-type-specific phenotype in response to the oncogenic potential of hsa_circ_0062682. This finding is in line with different classes of HCC tumours, and more studies are needed to shed a light on the molecular complexity of liver cancer.

## 1. Introduction

HCC represents the most common type of primary liver cancer and by the year 2025, the incidence of liver cancer is expected to reach more than 1 million individuals worldwide. Diverse aetiology is known for HCC with hepatitis B virus (HBV) and hepatitis C virus (HCV) infection currently representing the majority of cases, while metabolic-associated steatohepatitis (MASH) is an increasing aetiology, especially in the West. Since there are limited diagnostic, prognostic, and therapeutic approaches [1], a high morbidity rate is associated with this disease [2]. These aetiologies are also reflected in the different molecular pathogenesis of HCC [3]. Previous studies also identified different molecular subclasses of HCC, which are linked to different genetic and epigenetic alterations, enriched signalling pathways, immunological and histological changes, aetiologies, and clinical outcomes of the patients [4,5,6,7,8]. Additionally, liver cancer cell lines were also found to cluster in different molecular subclasses of HCC with different sensitivity to tested drugs [9].

CircRNAs are a diverse class of RNA molecules whose ends are covalently bound. They vary in function and they act by binding miRNAs and proteins, influencing gene expression, acting as a template for translation, etc. [10]. CircRNAs have been involved in pathological processes, such as in HCC, by being implicated in various tumour hallmarks, acting as potential oncogenes or tumour suppressors [11]. The majority of the dysregulated circRNAs in HCC have unknown roles and their importance in HCC pathogenesis needs to be evaluated. Different mechanisms contribute to the circRNA biogenesis such as the pairing of short interspersed nuclear repetitive DNA elements (SINEs) (especially Alu repetitive elements) in the intronic sequences [10], the presence of long flanking introns [12] and recently shown RNA editing [13,14]. The driving force of the differential expression of aberrantly expressed circRNAs in HCC remains unknown; however, regulators have already been proposed [15]. The stability of circRNAs and their tissue-specific expression [10] make them an interesting target for therapies. Additionally, circRNAs from extracellular fluids were found to have a good diagnostic potential in HCC patients [16].

In this study, we evaluated the oncogenic potential of an upregulated circRNA hsa_circ_0062682 in HCC. Since this circRNA was upregulated in published transcriptome data, we focused on the oncogenic potential in different HCC cell lines, the discovery of the underlying signalling pathways and transcription factors involved in the oncogenic phenotype in combination with the search for interaction partners using different omics technologies. 

## 2. Materials and Methods

### 2.1. Identification of Differentially Expressed circRNAs in HCC Tumours

Two microarray datasets from the GEO database [17], were used to identify circRNA expression levels of paired HCC and para-tumoral tissues (5 paired samples for GSE94508 [18] and 7 paired samples for GSE97332 [19]). Differential expression of circRNAs was analysed by GEO2R (http://www.ncbi.nlm.nih.gov/geo/geo2r/, accessed on 4 March 2019). Log transformation was applied prior to the analysis and *p*-values were adjusted for Benjamini and Hochberg’s false discovery rate (FDR). circRNAs with adjusted *p*-values ≤ 0.05 and fold change ≥ 1.5 or ≤−1.5 were marked as differentially expressed. Venn diagrams were drawn using the draw.io software. The visualization of the differential expression and fold changes was performed by GraphPad Prism 6 (GraphPad Software, San Diego, CA, USA).

### 2.2. Cell Lines

The Huh-7 cell line (300156, CLS, Eppelheim, Germany), SNU-499 cell lines (CRL-2234, ATCC, Manassas, VA, USA), and HepG2 cell line (Synthego Corporation, Menlo Park, CA, USA) were cultured in Dulbecco’s Modified Eagle’s Media (DMEM) high glucose (D6429, Sigma Aldrich, St. Louis, MO, USA), supplemented with 10% FBS (F0804, Sigma Aldrich, St. Louis, MO, USA) and 1% penicillin/streptomycin (P0781, Sigma Aldrich, St. Louis, MO, USA). Cells were cultured in a humidified chamber at a constant 37 °C and 5% CO_2_. 

### 2.3. Construction of a Modified Mini-Gene System for CircRNA Overexpression

To generate a mini-gene plasmid system for circRNA overexpression we modified a previously described mini-gene system pcDNA3.1(+) ZKSCAN1 MCS Exon Vector (69901, Addgene) [20], to enable overexpression of desired nucleotide sequence, without additional nucleotides at the site of the backsplice junction. By using the Q5 Site-Directed Mutagenesis Kit (E0554, New England Biolabs, Ipswich, MA, USA) we introduced mutations in the upstream and downstream regions of the mini-gene system splice sites for two restriction enzyme recognition sequences at sites 1392–1399 (ttttatac to atttaaat; *SwaI* recognition site) and 1464–1469 (agcaag to cgtacg; *BsiWI* recognition site) (Figure S1). The exact modified sequence and design of primers for cloning in the modified mini-gene system can be found in Table S1. We denoted the modified mini-gene system pRR. The sequence of hsa_circ_0062682 was amplified from the cDNA generated from the RNA of the Huh-7 cell line and cloned into the pRR plasmid (pRR-62682).

### 2.4. Transient Transfection

For transient overexpression, 500,000 Huh-7 cells were seeded on a 6-well plate 24 h before the transfection, to obtain 90–95% confluency on the day of the transfection. A total of 600,000 HepG2 cells were seeded on a collagen-treated 6-well plate 24 h before the transfection, to obtain 80–90% confluency on the day of the transfection. For the Huh-7 cell line, we used GenJet In Vitro DNA Transfection Reagent (SL100489-HUH, SignaGen Laboratories, Rockville, MD, USA) and followed the manufacturer’s protocol. For transient overexpression of HepG2 cell line, we used PolyJet In Vitro DNA Transfection Reagent (SL100688, SignaGen Laboratories, Rockville, MD, USA) and followed the manufacturer’s protocol while using 2 µg of plasmid DNA and 6 µL of PolyJet reagent per well.

For transient knockdown, 200,000 cells of the Huh-7 cell line were seeded on a 6-well plate 24 h before the transfection, to obtain approximately 50% confluency on the day of the transfection. For transient knockdown, we used PepMute siRNA Transfection Reagent (SL100566, SignaGen Laboratories, Rockville, MD, USA) and followed the manufacturer’s protocol by using 5 nM siRNA concentration.

### 2.5. Generation of Stable Cell Lines

For the generation of stable cell lines with overexpression or knockdown of hsa_circ_0062682, we used a lentiviral system. Transfer plasmid system pLKO.1–TRC cloning vector (10878, Addgene) was used for the cloning of the shRNA sequence (Table S1) and generating pLKO.1-62682.2 transfer plasmid. For negative control, we used scramble shRNA plasmid (1864, Addgene) derived from pLKO.1 plasmid. Transfer plasmid system pLenti-CMV-MCS-GFP-SV-puro (73582, Addgene) was used as a control to monitor transfection and transduction efficiency based on GFP expression. The sequence of hsa_circ_0062682 along with the intronic sequences and polyA tail was amplified from the pRR-62682 plasmid and cloned in the pLenti-CMV-MCS-GFP-SV-puro plasmid between XbaI and MluI restriction sites (pLenti-pRR-62682). A set of a control sequence containing an intronic sequence and poly A tail without the hsa_circ_0062682 sequence was amplified from the pRR plasmid and cloned in the same conditions (pLenti-pRR); 5 × 10^6^ HEK293T cells were seeded on 10 cm cell culture plates to obtain 80–90% confluency 24 h later. HEK293T cell line was transfected using 60 µL of PolyJet In Vitro DNA Transfection Reagent (SL100688, SignaGen Laboratories, Rockville, MD, USA), 10 µg of Ready-to-Use Lentiviral Packaging Plasmid Mix (CPCP-K2A, Cellecta, Mountain View, CA, USA) and 8 µg of transfer plasmid (pLenti-CMV-MCS-GFP-SV-puro, pLenti-pRR, pLenti-pRR-62682, scramble shRNA plasmid, pLKO.1-62682.2) were used following the manufacturer’s protocol. Six hours after the transfection medium was changed (DMEM supplemented with 10% FBS, penicillin/streptomycin and BSA (1.1 g/100 mL DMEM)); 72 h later the cell supernatant was collected and filtered through a 0.45 µm Millex Syringe Filter (SLHP033NS, Millipore, Burlington, MA, USA) and centrifuged at 150× *g*, 5 min. The supernatant was added to 30–40% confluent Huh-7 and SNU-449 cell line along with polybrene (8 µg/mL) (TR-1003-G, Sigma Aldrich, St. Louis, MO, USA) and the medium was changed the next day; 48 h later puromycin (508,838, Millipore, Burlington, MA, USA) was used to select stable cell lines.

### 2.6. RNase R Treatment

A total of 2 µg of isolated total RNA from the Huh-7 cell line was treated with RNase R (RNR07250, Epicentre Technologies, Madison, WI, USA) (4 U/µg of RNA) for 1 h at 37 °C and 20 min at 65 °C. Treated samples were reverse-transcribed by Maxima reverse transcriptase (EP0742, Thermo Scientific, Waltham, MA, USA) followed by RT-qPCR.

### 2.7. Cytoplasmic and Nuclear Localization of RNA and Proteins

Preparation of nuclear and cytoplasmic extracts was performed by the method of Schreiber et al. [21]. Cells from a 6-well plate were washed with PBS, scraped, and pelleted by centrifugation (150× *g*, 5 min) and resuspended in 300 µL cell lysis buffer (10 mM HEPES; pH = 7.5, 10 mM KCl, 0.1 mM EDTA, 1 mM DTT, 0.5% Nonidet-40, 40 U RNase OUT, cOmplete ULTRA tablets mini) for 20 min on ice with mixing. After vortexing, samples were centrifuged at 12,000× *g*, for 10 min at 4 °C to obtain cytoplasmic supernatants. Pelleted nuclei were washed three times in a cell lysis buffer (12,000× *g*, 10 min, 4 °C) and resuspended in nuclear extraction buffer (20 mM HEPES; pH = 7.5, 400 mM NaCl, 1 mM EDTA, 1 mM DTT, 40 U RNase OUT, and cOmplete ULTRA tablets mini) and incubated on ice for 30 min. Nuclear extracts were collected by centrifugation (12,000× *g*, 15 min, 4 °C). For RNA isolation, the supernatants were mixed with TRI reagent LS (T3934, Sigma Aldrich, St. Louis, MO, USA). For protein isolation, supernatants were mixed with 4 × Laemmli buffer (1,610,747, Bio-Rad Laboratories, Hercules, CA, USA). The specificity of subfractionation was further confirmed by antibody against Lamin A/C as a nuclear marker by Western blotting.

### 2.8. Actinomycin D Treatment

A total of 90–100% confluent Huh-7 cell line on a 6-well plate was treated with actinomycin D diluted in DMSO (A4262, Sigma-Aldrich, St. Louis, MO, USA) (2 µg/mL) or DMSO only as a control for 0, 4, 8, 12, and 24 h. The total RNA was isolated by TRI Reagent (T9424, Sigma Aldrich, St. Louis, MO, USA) and RNA expression levels were determined by RT-qPCR. Expression levels of measured RNA species were normalized to 18S rRNA expression levels.

### 2.9. Proliferation Assay

A total of 24 h post-transfection cell lines were trypsinised and counted with ADAM-MC Automated Cell Counter (NanoEntek, Seoul, Korea). A total of 3000 cells (Huh-7) and 8000 cells (HepG2) were seeded per well on 96-well plates in 4 replicates. Cell proliferation was measured by using Cell Counting Kit-8 (CK04, Dojindo Molecular Technologies, Rockville, MD, USA) following the manufacturer’s instructions. Absorbance at 450 nm was measured after 2 h of the incubation of the reagent with the cells on Epoch Microplate Spectrophotometer (Agilent Technologies, Santa Clara, CA, USA). All proliferation assays were performed as three independent experiments.

### 2.10. Wound Healing Assay

A total of 30,000–50,000 Huh-7 cells, 90,000 HepG2 cells, and 50,000 SNU-449 cells were seeded per a well in a Culture-Insert 2 well (80209, ibidi Technologies, Gräfelfing, Germany) in a 24-well plate; 24 h after, the culture insert was removed and cells were washed 2 times with PBS. Complete DMEM medium was added to each well and the wound area was imaged at indicated time points in the same areas. Cells were imaged on InCellis Cell Imager (Bertin Technologies SAS, Montigny-le-Bretonneux, France). The percentage of free wound area was calculated using the MRI Wound Healing Tool in ImageJ software.

### 2.11. Invasion Assay

At 48 h post-transfection cells were trypsinised and counted with ADAM-MC Automated Cell Counter (NanoEntek, Seoul, Korea); 24-well Corning BioCoat Matrigel Invasion Chambers, 8.0 µm PET Membrane (354480, Corning, Bedford, MA, USA), was used to investigate the invasion potential of cells. The test was conducted according to the manufacturer’s instructions. Briefly, 5 × 10^4^ cells in serum-free DMEM medium were added to the 24-well chambers and a complete DMEM medium (10% FBS) was added to the bottom of the wells. After 24 h, cell invasion was investigated by imaging on InCellis Cell Imager (Bertin Technologies SAS, Montigny-le-Bretonneux, France). Invading cells were stained with 0.5% crystal violet for 15 min. Six independent images were taken per well and the cell number was analysed using ImageJ software.

### 2.12. Colony Forming Assay

Huh-7, HepG2, and SNU-449 cell lines were counted and seeded on 6-well plates in triplicates and cultured for 8–15 days. Cells were then washed with PBS and fixated with 100% ice-cold methanol for 10 min. After fixation, cells were stained with 0.5% crystal violet for 15 min. Stained colonies were counted under the microscope using a manual cell counter. A variable number of cells were seeded per well depending on the experiment. The number of cells seeded per well is provided in the figure legends for each experiment.

### 2.13. EdU Assay

To investigate cell proliferation and percentage of cell population in the S phase of the cell cycle we used EdU-Click 555 (BCK-EdU555, baseclick GmbH, Munchen, Germany) following the manufacturer’s protocol. Cells were incubated with the reagent for 2 h. Cells were imaged on InCellis Cell Imager (Bertin Technologies SAS, Montigny-le-Bretonneux, France).

### 2.14. Immunostaining

For immunostaining cells were grown on µ-Dish 35 mm, high ibiTreat (81156, ibidi Technologies, Gräfelfing, Germany), washed with PBS and fixated by using 4% paraformaldehyde for 15 min. Cells were immunostained with Alexa Fluor 488 conjugated Ki-67 antibody D3B5 (1:50, Cell Signaling Technology, Danvers, MA, USA, 11882S) according to the manufacturer’s protocol. Cells were imaged on InCellis Cell Imager (Bertin Technologies SAS, Mon-tigny-le-Bretonneux, France).

### 2.15. Sorafenib Treatment

Cells were seeded at 5000 cells per well in 4 replicates on a 96-well plate and treated with sorafenib (SML2653, Sigma-Aldrich, St. Louis, MO, USA) diluted in DMSO 24 h after seeding. DMSO was used as a negative control; 24 h after starting the treatment cell viability was measured with Cell Counting Kit-8 (CK04, Dojindo Molecular Technologies, Rockville, MD, USA) by manufacturer’s instructions. Absorbance at 450 nm was measured after 2 h of the incubation of the reagent with the cells on Epoch Microplate Spectrophotometer (Agilent Technologies, Santa Clara, CA, USA).

### 2.16. Biotinylated Oligonucleotide Pulldown

Cells were grown to 90–95% confluency on 10 cm cell culture dishes, washed twice with ice-cold PBS, scraped, and collected. Cells were centrifuged at 150× *g*, for 5 min and PBS was aspirated. A total of 450 µL of Polysome Extraction Buffer (PEB) (20 mM Tris-HCl (pH = 7.5), 100 mM KCl, 5 mM MgCl2, 1 mM DTT, and 0.5% NP-40, supplemented with 100 U/mL RNase OUT (10777019, Invitrogen, Waltham, MA, USA)) and cOmplete ULTRA Tablets, Mini, EDTA-free (04693132001, Roche, Basel, Switzerland) per culture dish was added to the cells and pipetted up and down 10 times. Lysates were incubated on ice for 30 min and were vortexed every 10 min. After, incubation samples were centrifuged at 14,000× *g* for 30 min and 4 °C. After centrifugation the supernatant was mixed with 2 × TENT buffer (20 mM Tris-HCl (pH = 8.0), 2 mM EDTA (pH = 8.0), 500 mM NaCl, and 1% Triton X-100, supplemented with 100 U/mL RNase OUT and cOmplete ULTRA Tablets, Mini, EDTA-free) in a ratio 1:1. A total of 800 µL of prepared lysate was mixed by pipetting with 50 µL of Dynabeads M-280 (11205D, Invitrogen, Waltham, MA, USA) coupled with biotinylated oligonucleotides (Table S1) according to the manufacturer’s protocol. Coupled beads were incubated with lysates for 2 h at room temperature while rotating. After, the incubation beads were washed three times with 1 × TENT buffer. For protein analysis, beads were incubated with 30 µL of 1 × Laemmli buffer (1610747, Bio-Rad Laboratories, Hercules, CA, USA) at 95 °C for 5 min. For RNA analysis, beads were incubated with 500 µL of TRI Reagent (T9424, Sigma Aldrich, St. Louis, MO, USA) at room temperature for 10 min.

### 2.17. RNA Immunoprecipitation (RIP)

Cells were grown to 90–95% confluent on 10 cm cell culture dishes, washed twice with ice-cold PBS, scraped, and collected. Cells were centrifuged at 150× *g*, for 5 min and PBS was aspirated. A total of 500 µL of non-denaturing lysis buffer (20 mM TRIS-HCl (pH = 8.0), 137 mM NaCl, 2 mM EDTA, and 1% NP-40, supplemented with 100 U/mL Ribolock and cOmplete ULTRA Tablets, Mini, EDTA-free) per culture dish was added to the cells and pipetted up and down 10 times. Lysates were incubated on ice for 30 min and were vortexed every 5 min. After, incubation samples were centrifuged at 14,000× *g* for 30 min at 4 °C. Total protein concentration was measured by a Pierce BCA Protein Assay Kit (23225, Thermo Scientific, Waltham, MA, USA) and 280 µg of total protein was completed to 400 µL volume with non-denaturing lysis buffer. A total of 2 µg of YBX1 antibody (EP2708Y, Abcam, Cambridge, UK) or control rabbit IgG (12-370, EMD Millipore, Burlington, MA, USA) was added to 400 µL of a prepared lysate, mixed by pipetting and incubated at 4 °C for 2 h while rotating. Dynabeads Protein A (10002D, Invitrogen, Waltham, MA, USA) were prepared according to the manufacturer’s protocol and 50 µL of beads were added to a sample after incubation, followed by pipetting. Beads were incubated with the sample for 2 h at 4 °C while rotating. After, the incubation beads were washed three times with non-denaturing lysis buffer. For protein analysis, beads were incubated with 30 µL of 1 × Laemmli buffer (1610747, Bio-Rad Laboratories, Hercules, CA, USA) at 95 °C for 5 min. For RNA analysis, beads were incubated with 500 µL of TRI Reagent (T9424, Sigma Aldrich, St. Louis, MO, USA) at room temperature for 10 min.

### 2.18. RNA Isolation, Reverse Transcription and RT-qPCR

Total RNA was isolated by TRI reagent (T9424, Sigma Aldrich, St. Louis, MO, USA) or TRI Reagent LS (T3934, Sigma Aldrich, St. Louis, MO, USA) using the protocol provided by the manufacturer; 1–5 µg of total RNA was treated with DNase I (04716728001, Roche, Basel, Switzerland) and reverse-transcribed with random hexamers (SO142, Thermo Scientific, Waltham, MA, USA) using Maxima reverse transcriptase (EP0742, Thermo Scientific, Waltham, MA, USA) using the manufacturer’s protocol. cDNA was mixed with LightCycler 480 SYBR Green 1 Master (04707516001, Roche, Basel, Switzerland) and appropriate primers (300 nM concentration; Table S1). Touchdown RT-qPCR [22] was performed alongside negative controls (no reverse transcriptase and water) using a modified temperature program: 95 °C × 5 min for one cycle; 95 °C × 20 s and 66 °C × 10 s for 4 cycles by decreasing the annealing temperature 2 °C per cycle; 95 °C × 10 s, 60 °C × 10 s, and 72 °C × 10 s, for 45 cycles. The reactions were performed on the LightCycler 480 II instrument (Roche, Basel, Switzerland) and were run in technical triplicates. The obtained Ct values were normalized to the geometrical mean of beta-actin (*ACTB*) and 60S acidic ribosomal protein P0 (*RPLP0*) and the relative fold-change was determined using the 2^−ΔΔCt^ method.

### 2.19. Western Blotting

Proteins were resolved on Mini-PROTEAN TGX Stain-Free Precast Gels (4568043, Bio-Rad Laboratories, Hercules, CA, USA) and transferred to Immobilon-P PVDF Membrane (IPVH00010, Millipore, Burlington, MA, USA). Membranes were blocked for 1 h at room temperature with 5% milk/TBST. After blocking, membranes were incubated with primary antibodies overnight at 4 °C in recommended buffers (YBX1 (1:1000, Abcam, EP2708Y), IGF2BP1 (1:1000, CST, 8482), Mouse Anti-rabbit IgG (Conformation Specific) (1:2000, CST, 3678), hnRNP K (1:5000, Santa Cruz Biotechnology, Dallas, TX, USA, sc-28380), hnRNP C1/C2 (1:1000, Santa Cruz Biotechnology, sc-32308), CSNK2A1 (1:1000, Santa Cruz Biotechnology, sc-373894), ENO1 (1:1000, Santa Cruz Biotechnology, sc-101513), and Lamin A/C (1:1000, Santa Cruz Biotechnology, sc-376248). Membranes were washed four times with TBST for 5 min and incubated with secondary antibodies for 1 h at room temperature (goat anti-mouse IgG HRP (1:5000, Santa Cruz Biotechnology, sc-2005) and goat anti-rabbit IgG (H + L), HRP (1:20,000, Abcam, ab205718)). Membranes were washed four times with TBST for 5 min and imaged on ImageQuant LAS 4000 (GE Healthcare, Chicago, IL, USA) and iBright FL1500 Imaging System (Invitrogen, Waltham, MA, USA) by using Immobilon Classico Western HRP substrate (WBLUC, Millipore, Burlington, MA, USA) or SuperSignal West Femto Maximum Sensitivity Substrate (34094, Thermo Scientific, Waltham, MA, USA). Protein expression was normalized using the total protein normalization kit No-Stain Protein Labeling Reagent (A44449, Invitrogen, Waltham, MA, USA).

### 2.20. Silver Staining

Mini-PROTEAN TGX Stain-Free Precast Gels (4568043, Bio-Rad Laboratories, Hercules, CA, USA) were fixed for 2 h in fixation solution (50% methanol, 12% acetic acid, 0.05% formalin). Gels were washed three times for 20 min with 35% ethanol. After washing, gels were sensitized with 0.02% Na_2_S_2_O_3_ for 2 min and again washed three times for 5 min with dH_2_O. Gels were stained with staining solution (0.2% AgNO_3_, 0.076% formalin) for 20 min and washed two times for 1 min with dH_2_O. Gels were developed with developer solution (6% Na_2_CO_3_, 0.05% formalin, and 0.0004% Na_2_S_2_O_3_) and the staining was stopped with a stop solution (50% methanol, and 12% acetic acid) for 5 min. All steps were performed while rotating. Silver-stained gels were stored in 1% acetic acid at 4 °C.

### 2.21. Mass Spectrometry

Silver-stained protein bands were excised from the gel and destained as described previously [23]. Gel slices were subjected to reduction with 10 mM DTT in 25 mM ammonium bicarbonate, followed by alkylation with 55 mM iodoacetamide in the same buffer. Gel pieces were washed twice with 25 mM ammonium bicarbonate, dried on a speedvac and rehydrated in a 25 mM ammonium bicarbonate containing 1 ug of porcine sequence grade modified trypsin (V5111 Promega, Madison, WI, USA). Samples were digested overnight at 37 °C. Digested peptides were extracted from the gel with 50% acetonitrile solution containing 5% formic acid and concentrated to 15 µL. Samples were analysed with LTQ Orbitrap Velos mass spectrometer (Thermo Scientific, Waltham, MA, USA) coupled to a Proxeon-nanoLC (Proxeon, Odense, Denmark) liquid chromatography unit. Peptides were loaded on a C18 EASY trapping column (SC001 Proxeon, Odense, Denmark) and separated on a C18 PicoFritTM AQUASIL analytical column (PF7515-100H053 New Objective, Littelton, MA, USA) with a flow rate of 300 nL/min. Elution was performed with a 60 min acetonitrile gradient from 5 to 40% in the 0.1% solution of formic acid, with a flow rate of 300 nL/min. The nine most intense precursor ions in each full scan were selected for HCD fragmentation. The dynamic exclusion was set at a repeat count of 1 with an exclusion duration of 60 s. Database searches were performed against the human NCBInr database using the Mascot search algorithm (Matrix Science, London, UK) integrated into the Proteome Discoverer software package (Thermo Scientific, Waltham, MA, USA). Carbamidomethylation of cysteins was set as fixed and oxidation of methionines as a dynamic modification. Scaffold (Proteome Software Inc., Portland, OR, USA) was used to validate MS/MS-based peptide and protein identifications. Peptide identifications were accepted if they were identified at least by 95.0% probability using the Peptide Prophet algorithm [24]. Protein identifications were accepted if they were identified by at least 99.9% probability and with at least 2 identified peptides. Protein identification probabilities were assigned by the Protein Prophet algorithm [25]. We excluded potential contaminants such as cytokeratins, tubulins, actins, and ribosomal proteins [26] from further analyses. Proteins were ranked by normalized total spectra.

### 2.22. Functional Protein Network Analysis

Proteins identified by mass spectrometry by at least two unique peptides were taken into the analysis in a STRING database [27] (https://string-db.org/, accessed on 12 September 2021) by a minimum required interaction score set to the highest confidence (0.900).

### 2.23. Identification of Differentially Expressed Genes in HCC

Differential expression of mRNA in HCC was analysed in R using the TCGA-Biolinks package (accessed on 22 June 2020; [28]) from the TCGA-LIHC cohort (371 tumour samples and 50 normal samples). Normalization using EDASeq protocol was used to apply between-lane normalization and within-lane normalization (accounting for differences in gene length). Pre-processing and filtering of data were performed as suggested. *p*-values were adjusted for Benjamini and Hochberg’s false discovery rate (FDR). mRNA with adjusted *p*-value ≤ 0.01 was marked as differentially expressed. There was no cut-off value for fold change in analyses. The visualization of the differential expression from the TCGA-LIHC cohort in the TCGA dataset was performed by GraphPad Prism 6 (GraphPad Software, San Diego, CA, USA).

### 2.24. Survival Analysis

Overall survival analysis was analysed by Kaplan–Meier analysis using a KM plotter for the TCGA-LIHC cohort (https://kmplot.com/analysis/, accessed on 11 October 2021) [29]. Patients were divided into high- and low-expression groups according to the median gene expression. Associations with log-rank *p*-values ≤ 0.05 were marked as significant. 

### 2.25. Gene Expression Profiling, Gene Set Enrichment Analysis and Pathway Enrichment Analysis

Transcriptome analysis of HCC model cell lines (transient overexpression in Huh-7 and stable knockdown in SNU-449 with appropriate controls) was performed using Clariom S Array, human (902917, Applied Biosystems, Waltham, MA, USA) in triplicates from each cell line according to manufacturer’s protocol. The data were analysed in Transcriptome Analysis Console (TAC) and FDR ≤ 0.05 was used as a cut-off to identify differentially expressed genes in SNU-449 cell lines, whereas unadjusted *p*-value ≤ 0.05 and average log2 expression value ≥ 6 were used as a cut-off to identify differentially expressed genes in Huh-7 cell lines. 

Pathway enrichment analysis was performed according to Reimand et al. 2019 [30], using GSEA, Cytoscape and EnrichmentMap tools. Q value ≤ 0.1 was used to identify enriched pathways in SNU-449 cell lines and Q value ≤ 0.25 was used to identify enriched pathways in Huh-7 cell lines. Gene set enrichment analysis of KEGG pathways and TRRUST transcription factors in differentially expressed genes was performed by Enrichr [31].

### 2.26. Statistical Analysis

To compare pairs of independent groups Student’s *t*-test was used. Statistical analysis and visualization were performed with GraphPad Prism 6 (GraphPad Software, San Diego, CA, USA). Results were presented as the averages and error bars represent standard deviation. *p*-values ≤ 0.05 were considered significant. The significant levels were defined as: * *p* < 0.05, ** *p* < 0.01, *** *p* < 0.001, and **** *p* < 0.0001.

## 3. Results

### 3.1. CircRNA hsa_circ_0062682 Is Upregulated in Hepatocellular Carcinoma

**Figure 1:**
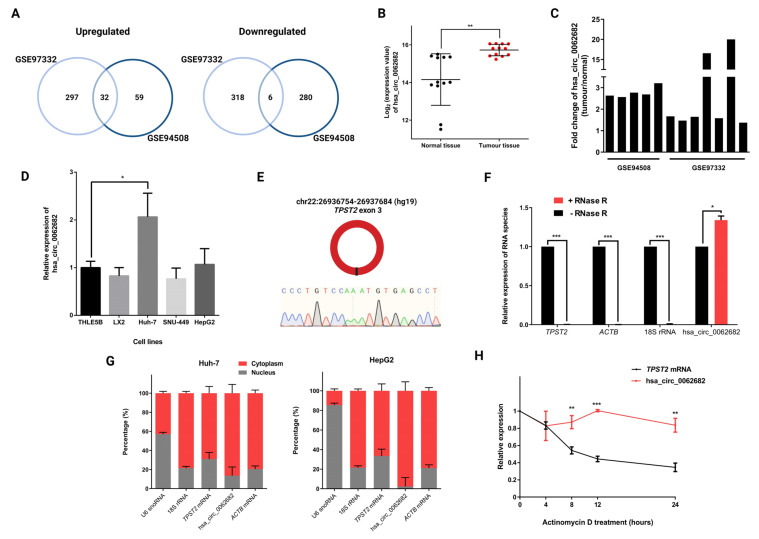
Hsa_circ_0062682 is upregulated in HCC and is predominantly localized in the cytoplasm. (**A**) Venn diagrams showing differentially expressed circRNAs found in two studies (GSE94508, GSE97332) (fold change (FC) ≥ 1.5 or ≤−1.5; adjusted *p*-value ≤ 0.05); (**B**) log_2_ of hsa_circ_0062682 expression in normal tissues and tumour tissues in both studies; (**C**) fold change in hsa_circ_0062682 expression in paired tumour tissue vs. normal tissue in patients of aforementioned studies; (**D**) relative expression of hsa_circ_0062682 in liver model cell lines, normalized to the geometric mean of *ACTB*/*RPLP0* expression (n = 3); (**E**) schematic representation of a single exon circRNA hsa_circ_0062682 and its backsplice sequence; (**F**) relative expression of RNA species after treating 1 µg of Huh-7 total RNA with RNase R (n = 3); (**G**) localization of hsa_circ_0062682 and other RNA species in Huh-7 (left) (n = 3) and HepG2 (right) cell lines (n = 3); U6 snoRNA served as a positive control for the nuclear fraction and 18S rRNA served as a positive control for the cytoplasmic fraction; (**H**) relative expression of hsa_circ_0062682 and *TPST2* mRNA after treatment of Huh-7 cell line with actinomycin D (n = 3). results are represented as averages and error bars represent standard deviation. * *p*-value ≤ 0.05; ** *p*-value ≤ 0.01; *** *p*-value ≤ 0.001; Student’s *t*-test.

To obtain differentially expressed circRNAs in HCC we used two published datasets (GSE94508 [18] and GSE97332 [19]), which compared circRNA expression in tumour samples vs. adjacent normal tissue samples in five patients and seven patients, respectively. By using the GEO2R tool (adjusted *p*-value ≤ 0.05; fold change ≥ 1.5 or ≤−1.5) we identified 32 upregulated and 6 downregulated circRNAs common in both studies (Figure 1A, Table S2). Among identified differentially expressed circRNAs, hsa_circ_0062682 was shown to be upregulated in tumour samples compared with normal tissue samples (Figure 1B,C) and was also one of the most upregulated circRNAs in HCC model cell line HepG2 in a published study [32]. Additionally, it was found to be upregulated in the Huh-7 HCC model cell line in comparison to immortalized hepatocyte cell line THLE5B (Figure 1D). Hsa_circ_0062682 is derived from a single exon 3 of the *TPST2* gene and is comprised of 930 nucleotides (Figure 1E). Its circular structure was further confirmed upon treatment with RNase R where the expression level of hsa_circ_0062682 was not decreased as other linear RNA species were (Figure 1F). In HCC model cell lines, Huh-7 and HepG2, it was shown to be predominantly localized in the cytoplasm (Figure 1G) and was shown to be very stable upon actinomycin D treatment with half-life extending the 24 h period of measurement unlike the mRNA from the same gene locus (*TPST* mRNA t1/2 ≈ 8 h) (Figure 1H).

### 3.2. Perturbation of hsa_circ_0062682 Affected the Oncogenic Potential of HCC Cell Line Models

**Figure 2:**
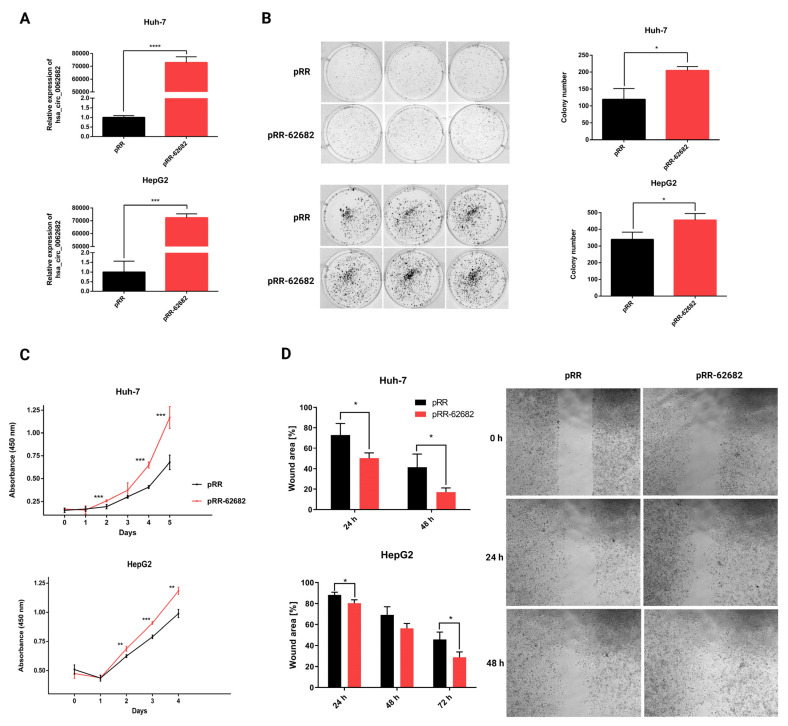
Transient overexpression of circular RNA hsa_circ_0062682 promoted the oncogenic potential of HCC model cell lines. (**A**) Relative expression of hsa_circ_0062682 in transiently overexpressing Huh-7 (**up**) (n = 3) and HepG2 (**down**) cell line (n = 3); (**B**) colony forming assay in hsa_circ_0062682-overexpressing Huh-7 (left) (n = 3) (10,000 cells) and HepG2 (**right**) cell lines (n = 3) (5000 cells); (**C**) proliferation of hsa_circ_0062682-overexpressing Huh-7 (**up**) (n = 4) and HepG2 (**down**) cell lines (n = 4); (**D**) wound healing assay of hsa_circ_0062682-overexpressing Huh-7 (**up**) (n = 4) and HepG2 (**down**) (n = 4) cell lines; representative images of Huh-7 migration are shown on the right; representative images of HepG2 migration can be found in Supplementary Materials; pRR–empty plasmid, pRR-62682–plasmid for hsa_circ_0062682 overexpression; results are represented as averages and error bars represent standard deviation. * *p*-value ≤ 0.05; ** *p*-value ≤ 0.01; *** *p*-value ≤ 0.001; **** *p*-value ≤ 0.0001, Student’s *t-*test.

To investigate the oncogenic potential of the upregulated hsa_circ_0062682 we modified a previously described plasmid mini-gene system (pcDNA3.1(+) ZKSCAN1 MCS Exon Vector, [20]) to enable overexpression of the desired cloned circRNA sequence without the presence of additional nucleotides in the backsplice junction in the transcribed RNA sequence and denoted it pRR (Methods section, Supplementary Materials Figure S1). Transient overexpression of hsa_circ_0062682 in HCC model cell lines Huh-7 and HepG2 (Figure 2A) significantly increased the number of colonies in the colony-forming assay (Figure 2B). By performing a colourimetric CCK-8 assay we observed increased cell proliferation (Figure 2C) in overexpressed cells. The wound healing assay additionally confirmed its oncogenic potential in overexpressed cells which migrated faster (Figure 2D and Figure S2).

**Figure 3:**
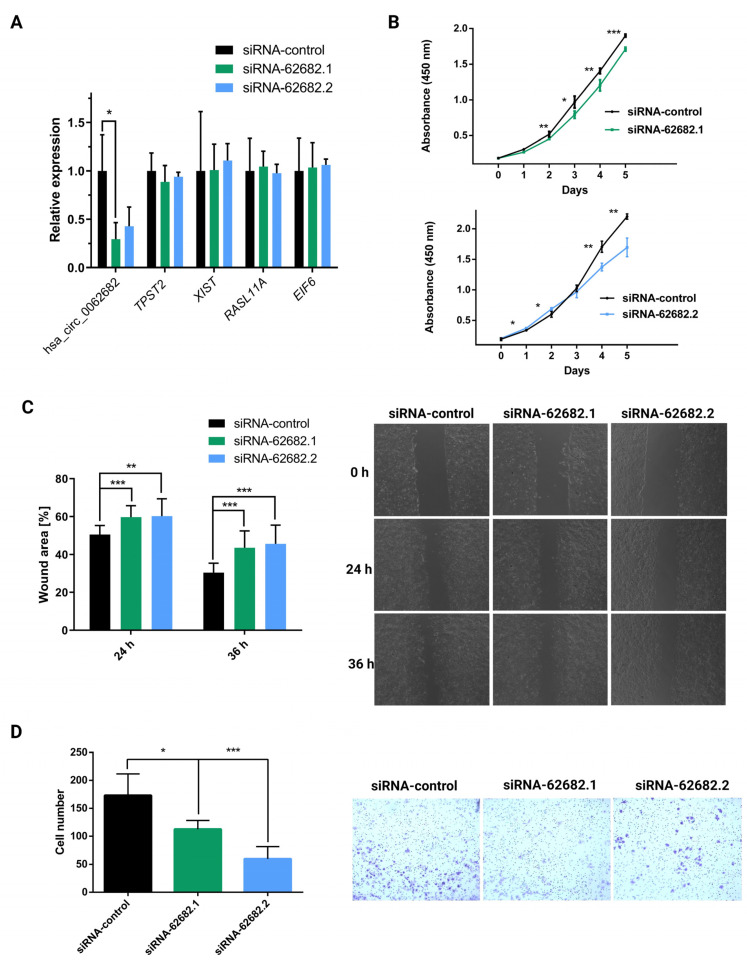
Transient knockdown of circular RNA hsa_circ_0062682 by two different siRNA sequences (siRNA-62682.1, siRNA-62682.2) decreased the oncogenic potential of the Huh-7 model cell line. (**A**) Relative expression of hsa_circ_0062682 and potential off-targets identified by nucleotide BLAST in Huh-7 cell line (n = 3); (**B**) proliferation of Huh-7 cell line with a knockdown of hsa_circ_0062682 (n = 4); (**C**) wound healing assay of Huh-7 cell line with a knockdown of hsa_circ_0062682 (n = 12); on the right representative images of Huh-7 migration are shown; (**D**) invasion assay of Huh-7 cell line with a knockdown of hsa_circ_0062682 (n = 18). Results are represented as averages and error bars represent standard deviation. * *p*-value ≤ 0.05; ** *p*-value ≤ 0.01; *** *p*-value ≤ 0.001, Student’s *t*-test.

Since the Huh-7 cell line has the highest expression of hsa_circ_0062682, we knocked down its expression by using two different siRNAs (Table S1), which showed no knockdown effect on potential off-targets (Figure 3A). Transient knockdown of hsa_circ_0062682 significantly decreased cell proliferation (Figure 3B), migration (Figure 3C), and cell invasion (Figure 3D), further confirming its oncogenic potential in the HCC model cell line Huh-7.

**Figure 4:**
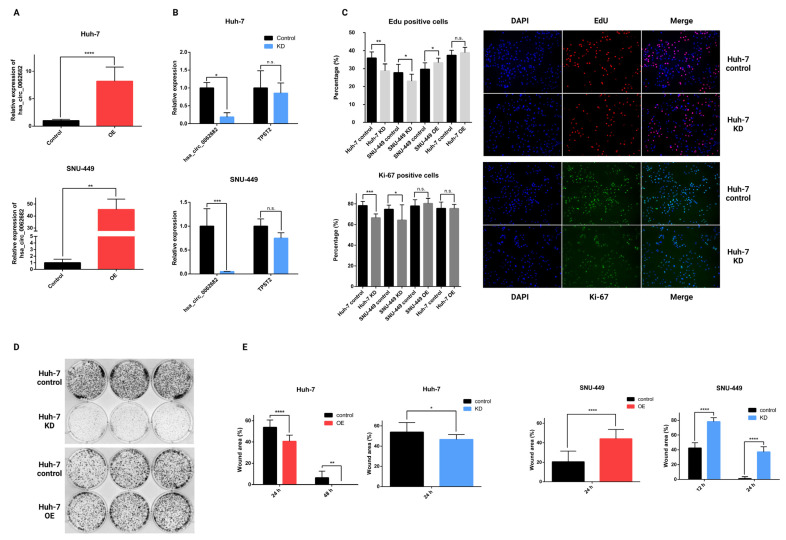
Stable overexpression (OE) and knockdown (KD) of hsa_circ_0062682 in HCC model cell lines. (**A**) Relative expression of hsa_circ_0062682 in overexpressing Huh-7 (n = 3) and SNU-449 (n = 3) cell lines; (**B**) relative expression of hsa_circ_0062682 and TPST2 in a knockdown of hsa_circ_0062682 in Huh-7 (n = 3) and SNU-449 (n = 3) cell lines; (**C**) EdU proliferation assay (n = 12) (**top**) and Ki-67 immunostaining (n = 12) (**bottom**) of stably transduced model cell lines Huh-7 and SNU-449; representative images of Huh-7 knockdown cell line and its representative control are shown on the right (magnification = 200×); (**D**) Colony forming assay in transduced Huh-7 cell lines (10,000 cells); (**E**) wound healing assay of overexpressing Huh-7 and SNU-449 (n = 11) and in a knockdown of Huh-7 and SNU-449 cell lines (n = 11); representative images can be found in Supplementary Materials (Figures S4 and S5); OE: overexpression; KD: knockdown. Results are represented as averages and error bars represent standard deviation. * *p*-value ≤ 0.05; ** *p*-value ≤ 0.01; *** *p*-value ≤ 0.001; **** *p*-value ≤ 0.0001; n.s.: not significant, Student’s *t*-test.

To further investigate the role of hsa_circ_0062682 in HCC, we decided to include another HCC model cell line SNU-449, which represents a different subtype of HCC. Huh-7 represents a subtype expressing hepatocyte and liver progenitor markers and SNU-449 represents a less differentiated, more invasive subtype that expresses a higher level of epithelial-to-mesenchymal transition (EMT) and stem cell markers and lower levels of hepatocyte-specific markers [9]. Next, we generated stable Huh-7 and SNU-449 cell lines overexpressing hsa_circ_0062682 by using the lentiviral transduction (Figure 4A). Furthermore, we generated a stable knockdown of hsa_circ_0062682 (Figure 4B). By conducting an EdU incorporation assay (Figure 4C), we confirmed the role of hsa_circ_0062682 in cell proliferation where its knockdown in Huh-7 and SNU-449 cell lines resulted in a lower EdU signal indicating that fewer cells were in the S phase of the cell cycle. By performing the Ki-67 immunostaining, we detected significantly fewer positive cells in the knockdowns of both cell lines, indicating that the knockdown of hsa_circ_0062682 promoted the resting G0 phase in these cell lines (Figure 4C). Moreover, overexpression of hsa_circ_0062682 in the SNU-449 cell line resulted in more positive cells for the EdU signal compared with the control. Colony-forming assay indicated a higher clonogenic potential in overexpressing cell lines (Figure 4D and Figure S3) and a lower clonogenic potential in knockdown cell lines (Figure 4D and Figure S3). Wound healing assays in overexpressing Huh-7 cell line confirmed the role of hsa_circ_0062682 in promoting the migration of model cell lines, while knockdown of hsa_circ_0062682 in Huh-7 cell line also unexpectedly promoted migration (Figure 4E and Figure S4). In contrast, knockdown and overexpression of hsa_circ_0062682 in SNU-449 cell line decreased cell migration (Figure 4E and Figure S5), suggesting other cell-type-specific factors play a crucial role in hsa_circ_0062682-mediated impact on cell migration in stable cell lines.

### 3.3. Modulation of the hsa_circ_0062682 Expression Induced Changes in the Transcriptome of HCC Model Cell Lines

**Figure 5:**
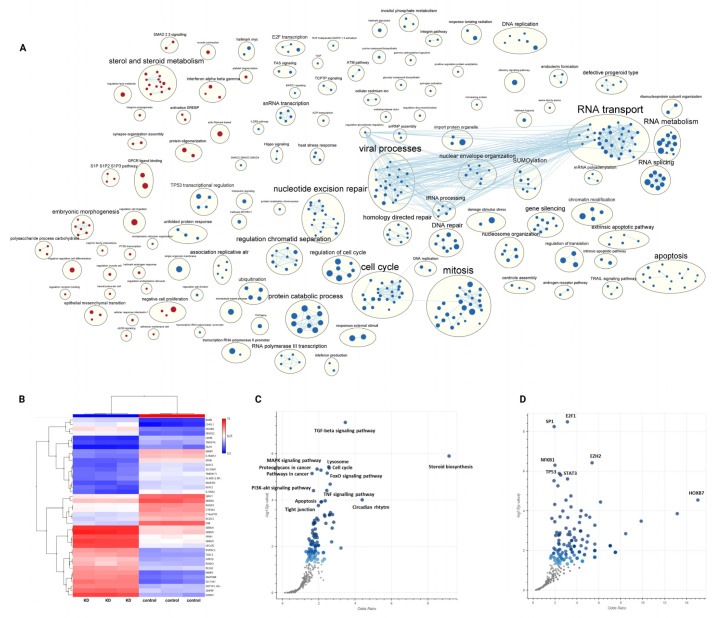
Knockdown of hsa_circ_0062682 in SNU-449 cell line induces profound changes on the transcriptomic level. (**A**) Pathway enrichment analysis of upregulated (red colour) and downregulated (blue colour) pathways. Each node (circle) represents a pathway and edges represent shared genes by the connected pathways. Annotations of clusters of similar pathways representing major biological themes are provided above clusters; (**B**) heatmap of top 20 most upregulated and top 20 most downregulated genes in a stable KD of hsa_circ_0062682 in SNU-449 cell line, heatmap legend consists of log2 transformed expression values; (**C**) volcano plots from Enrichr analysis of enriched gene sets in KEGG pathways and in (**D**) the TRRUST database of transcription factors, made by Appyter. Blue dots represent significantly enriched gene sets, grey dots represent not enriched gene sets.

To better understand the molecular mechanisms and systemic changes upon which hsa_circ_0062682 acts in HCC model cell lines, we analysed the transcriptome of a stable knockdown in the SNU-449 cell line and transient overexpression in the Huh-7 cell line and their respective controls using microarrays. We identified 1717 upregulated and 1546 downregulated genes (FDR ≤ 0.05; Table S3, Figure S6) in the knocked down SNU-449 cell line. A heatmap of the expression of the top 20 most upregulated and most downregulated genes (by fold-change) between the two groups is shown in Figure 5B. By performing pathway enrichment analysis [30] we identified several enriched processes (Figure 5A, Table S4). Namely, cell processes connected with cell proliferation (mitosis, cell cycle, and DNA replication), DNA repair, apoptosis, unfolded protein response, RNA splicing, transport, and metabolism were found to be downregulated, while processes such as steroid metabolism, morphogenesis and the GPCR ligand binding were found to be upregulated. In order to compare the changes in the transcriptome observed in a stable knockdown in the SNU-449 cell line with a transient overexpression in the Huh-7 cell line, we had to use a less strict *p*-value for identification of differentially expressed genes in the analysis of transcriptome data in Huh-7 cell line (620 upregulated, 398 downregulated; *p*-value ≤ 0.05; Table S3). Nevertheless, we confirmed the differential expression for selected genes by RT-qPCR (Figure S7) and observed the opposite enrichment of several pathways in comparison to knockdown, namely negative enrichment of steroid metabolism and the GPCR ligand binding and the positive enrichment of RNA splicing and unfolded protein response (Figure S8, Table S4).

Gene set enrichment analysis (GSEA) of differentially expressed genes in knocked down SNU-449 cells in Enrichr [31] showed enrichment of multiple KEGG pathways (Table S5, Figure 5C). Several signalling pathways implicated in the oncogenesis of HCC (TGF-beta [33,34], MAPK [35], PI3K-Akt [36,37], FoxO [38], and TNF signalling pathway [39,40]) were found to be among the most enriched processes. Identified enriched signalling pathways involved in HCC pathology can explain the effects observed in cell-based assays for cell proliferation, migration, and invasion. Among the most enriched KEGG pathways in GSEA analysis were also cell cycle, apoptosis, and steroid biosynthesis, which all were confirmed by the pathway enrichment analysis. The involvement of circadian rhythm in HCC has also been previously described [41]. GSEA of differentially expressed genes in the knocked down SNU-449 in Enrichr also identified several enriched transcription factors from the TRRUST database [42], of which the most enriched were E2F1, SP1, EZH2, NFKB1, TP53, and STAT3 (Table S5, Figure 5D). By separating differentially expressed genes into upregulated and downregulated sets we could observe an enrichment of some KEGG pathways only in a set of downregulated genes (cell cycle) or in a set of upregulated genes (lysosome, steroid biosynthesis, FoxO signalling pathway, etc.) (Table S6). Likewise, we could observe the separation of enriched transcription factors (Table S7, Figure S9) in a set of downregulated genes (E2F1, SP1, HIF1A, YBX1, etc.) and a set of upregulated genes (NFKB1, EZH2, TWIST2, RELA, etc.) suggesting activation of some transcription factors and repression of others by hsa_circ_0062682 perturbation.

### 3.4. Hsa_circ_0062682 Binds to YBX1 and Its Effect Is Cell-Type Specific

Numerous previous studies have shown that circRNAs can exert their functions by binding to and acting on proteins, especially RNA-binding proteins (RBPs) by functioning as a sponge, decoy, or scaffold [43,44]. To understand the mechanism by which the hsa_circ_0062682 exerts its oncogenic potential in HCC we wanted to investigate potential protein binding partners. We first performed the in silico analyses of potential binding to RNA-binding proteins using different RBP binding prediction algorithms: RBPmap [45], CISBP-RNA [46], and RBPDB [47]. When comparing results from the in silico analyses, we detected several RBPs that were found by at least two algorithms (Table S8), indicating the potential for hsa_circ_0062682 to bind RBPs.

**Figure 6:**
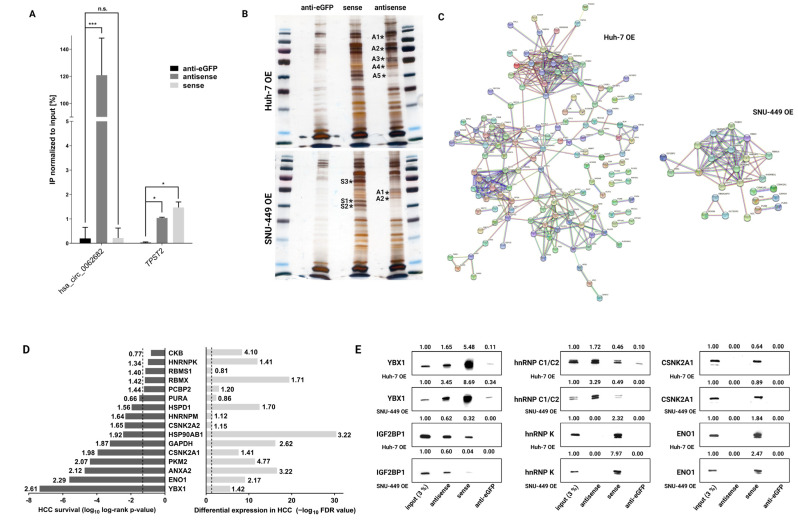
Biotinylated oligonucleotide pulldown of hsa_circ_0062682 coupled with mass spectrometry identifies its potential protein binding partners. (**A**) Confirmation of a specific pulldown by RT-qPCR (n = 3) in Huh-7 OE cell line; (**B**) silver staining of biotinylated oligonucleotide pulldowns in Huh-7 OE (**top**) and SNU-449 OE (**bottom**) cell lines (* indicates excised bands for mass spectrometry analysis; letters and numbers indicate excised bands indicated in Table S9); (**C**) functional protein network analysis in STRING database of identified proteins by mass spectrometry (minimum 2 unique peptides present); (**D**) alignment of top identified proteins by mass spectrometry with differential expression in HCC (**right**: numbers indicate fold change ratio between the tumour and normal samples) and with overall survival of HCC patients (**left**: numbers indicate hazard ratio); dotted line indicates *p*-value ≤ 0.05; (**E**) Western blotting of potential protein binding partners in biotinylated oligonucleotide pulldowns of both stable overexpressing cell lines; numbers indicate densitometric analysis of presented bands normalized to the input. OE: overexpression. Results are represented as averages and error bars represent standard deviation. The uncropped blots are shown in Supplementary Data.

Next, we wanted to experimentally identify the potential interactions of hsa_circ_0062682 with RNA-binding proteins and performed biotinylated oligonucleotide pulldown of hsa_circ_0062682 coupled with mass spectrometry. We performed the pulldown assay on stably overexpressing Huh-7 and SNU-449 cell lines. A 32-nucleotides antisense oligonucleotide was designed to base-pair with the backsplice junction sequence of the hsa_circ_0062682, as well as the 32-nucleotides long sense oligonucleotide, which represents the backsplice junction, and an anti-eGFP oligonucleotide of the same length and GC content which was used as a control. Biotinylated oligonucleotide pulldown followed by RT-qPCR confirmed a significant enrichment of hsa_circ_0062682 in samples treated with antisense oligonucleotide in comparison to samples treated with sense or anti-eGFP oligonucleotide (Figure 6A). By silver staining of gels, we identified several enriched bands in the antisense and sense biotinylated oligonucleotide pulldowns in both cell lines compared with the anti-eGFP biotinylated oligonucleotide pulldowns (Figure 6B). Mass spectrometry analysis of enriched bands revealed a total of 81 proteins in the analysed samples from the SNU-449 cell line and 219 proteins from the Huh-7 cell line (threshold ≥ 2 peptides) (Table S9) and constructed functional protein networks using the STRING database [27] (Figure 6C). The constructed networks from both cell lines had a significant PPI (protein–protein interaction) enrichment *p*-value (<1 × 10^−16^). In both protein networks, we found significantly enriched two Reactome pathways, mRNA splicing and RNA metabolism (Table S10) which were also perturbed in the pathway enrichment analyses of the transcriptomes of model HCC cell lines (Figure 5B and Figure S8, Table S4), indicating hsa_circ_0062682 can act by binding RBPs which modulate RNA metabolism and splicing.

We additionally aligned the top 2 identified proteins from each excised band (Figure 6B) with the HCC survival data and differential expression of HCC patients from the TCGA-LIHC dataset (Figure 6D). Most of the analysed proteins, which are known to act as RBPs, were significantly upregulated in HCC and were negatively correlated with the overall survival of HCC patients. This furthermore suggests the implication of hsa_circ_0062682 in the oncogenesis of HCC.

We next focused on the interaction of the hsa_circ_0062682 with proteins previously implicated in the pathology of HCC, namely YBX1 (Y-box-binding protein 1) [48], IGF2BP1 (Insulin-like growth factor 2 mRNA-binding protein 1) [49], hnRNP C1/C2 (Heterogeneous nuclear ribonucleoproteins C1/C2) [50], hnRNP K (Heterogeneous nuclear ribonucleoprotein K) [51], CSNK2A1 (Casein kinase II subunit alpha) [52], and ENO1 (Alpha-enolase) [53]. While YBX1, IGF2BP1, hnRNP C1/C2, and hnRNP K are known to bind RNAs and have roles in RNA metabolism, it has also been shown for CSNK2A1 and ENO1 to be able to bind RNA molecules [54,55,56]. The binding of proteins was further evaluated by biotinylated oligonucleotide pulldown followed by Western blotting (Figure 6E). We confirmed the signal enrichment of YBX1, IGF2BP1, and hnRNP C1/C2 in the samples pulled down by antisense oligonucleotide indicating their binding to hsa_circ_0062682 (Figure 6E). Interestingly, we also detected signal enrichment in all the tested proteins in the samples pulled down by the sense oligonucleotide which represents the backsplice junction of hsa_circ_0062682 which can indicate the binding site of identified proteins is in the proximity of the backsplice junction. 

**Figure 7:**
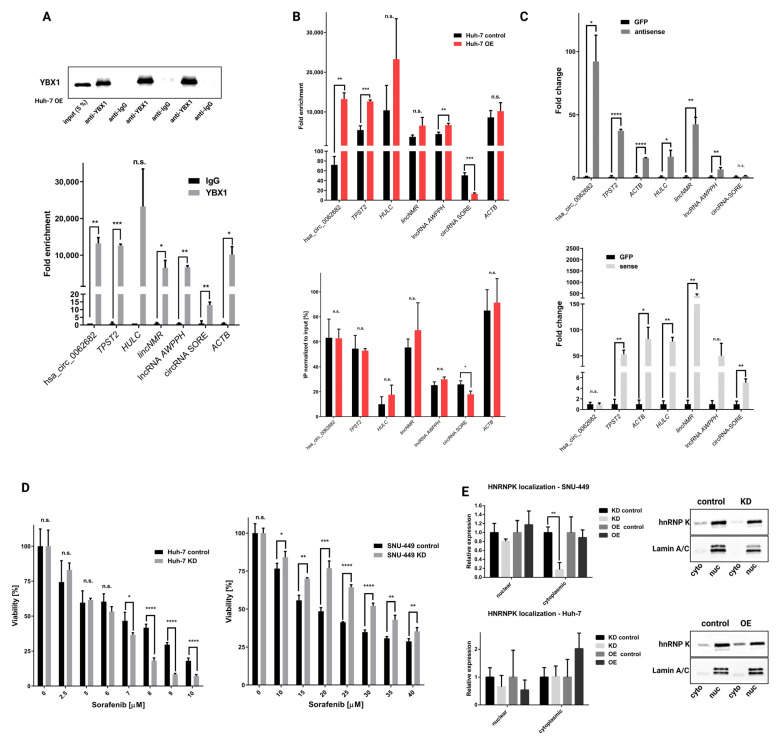
Hsa_circ_0062682 binds to YBX1 and its effect is cell-context dependent. (**A**) Confirmation of a specific pulldown from RNA immunoprecipitation of YBX1 by Western blotting (up); enrichment of RNA species from RNA immunoprecipitation by RT-qPCR (n = 3) (down); (**B**) comparison of RNA immunoprecipitation enrichment between Huh-7 OE and Huh-7 control cell line normalized to expression levels in IgG fractions (top) or expression levels in the input (bottom) (n = 3); (**C**) enrichment of RNA species in biotinylated oligonucleotide pulldowns normalized to biotinylated anti-GFP oligonucleotide pulldown; antisense (up), sense (down) (n = 3); (**D**) viability of sorafenib treated KD cell lines measured by colourimetric CCK-8 assay (n = 4); (**E**) hnRNP K localization in hsa_circ_0062682 stable cell lines identified by Western blotting (n = 3); OE: overexpression; KD: knockdown; cyto: cytoplasmic fraction; nuc: nuclear fraction. Results are represented as averages and error bars represent standard deviation. * *p*-value ≤ 0.05; ** *p*-value ≤ 0.01; *** *p*-value ≤ 0.001; **** *p*-value ≤ 0.0001; n.s.: not significant, Student’s *t*-test. The uncropped blots are shown in Supplementary Data.

Taking into consideration several results from our study, we decided to focus on the interaction between hsa_circ_0062682 and YBX1: (1) in silico analysis of hsa_circ_0062682 protein binding partners identified 4 binding sites by two algorithms; (2) YBX1 was one of the most abundant proteins in the biotinylated oligonucleotide pulldown, identified by mass spectrometry; (3) expression of YBX1 is significantly upregulated in HCC, and (4) expression of YBX1 is negatively correlated to the overall survival of HCC patients. Next, we performed RNA immunoprecipitation using the YBX1 antibody followed by Western blotting to confirm the specificity of our pulldown (Figure 7A). CircRNA hsa_circ_0062682 as well as other RNA molecules, which have been previously described to bind to YBX1 [57], also in the context of HCC [58,59,60,61], were found to be enriched in the pulled down fractions followed by RT-qPCR in comparison to IgG negative control (Figure 7A). When we compared pulldowns between stably overexpressing cell line Huh-7 and its control, we observed a significant enrichment of RNA molecules bound to YBX1 (Figure 7B). However, when normalizing to the input in stably overexpressing and in the control cell lines, only circRNA-SORE was shown to be significantly less abundant in the YBX1 pulled down samples of overexpressing cell line (Figure 7B). This result can indicate a possible competing mechanism between the two circular RNAs. We further measured the enrichment of other RNA species in the biotinylated oligonucleotide pulldown, followed by RT-qPCR. Interestingly, RNA species that were shown to be enriched in YBX1 fractions of the RNA immunoprecipitation experiment were also enriched in the antisense oligonucleotide pulldown samples except for circRNA-SORE (Figure 7C and Figure S10). Conversely, circRNA-SORE was shown to be enriched in the sense oligonucleotide fractions where hsa_circ_0062682 was not found to be enriched, again implicating a possible competing mechanism between the two circRNAs in binding to the YBX1. Since YBX1 was already implicated in sorafenib resistance in HCC [62], also in the context of binding to circRNA-SORE [61], we also investigated the effect of sorafenib on the viability of our stable cell lines (Figure 7D and Figure S11). The observed effect was most profound in KD cell lines; however, it was not consistent among Huh-7 and SNU-449 KD cell lines. Huh-7 KD cell line was found to be profoundly more sensitive to the sorafenib treatment while the SNU-449 KD cell line was more resistant. Observed differences may also be due to the fact that circRNA-SORE is differentially expressed in HCC and liver model cell lines (Figure S12), implicating a possibly more complex interplay between hsa_circ_0062682, circRNA-SORE and YBX1. We additionally investigated localization for some of the pulled down proteins, namely YBX1 and hnRNP K. It is well-known that their role is influenced by the nuclear and cytoplasmic ratio [63,64,65]. While YBX1 did not show any changes in the localization in both model cell lines (Figure S13), hnRNP K was less present in the KD SNU-449 cell line (Figure 7E) while no apparent changes were seen in the Huh-7 KD cell line. Cytoplasmic accumulation of hnRNP K was previously connected to the process of cell motility [64] and is in concordance with the wound healing assay (Figure 4E). MAPK signalling pathway which plays a role in the cytoplasmic accumulation of hnRNP K [63] was also found to be enriched by the GSEA analysis in the SNU-449 KD cell line (Figure 5C). Results obtained from the wound healing assay, sorafenib treatment, and localization assay suggest that hsa_circ_0062682 plays a different role depending on a cell type and possibly an HCC-subtype context.

## 4. Discussion

Numerous studies have shown an important role of non-coding RNAs in the pathology of different oncological diseases, also in HCC. The class of circRNAs, firstly viewed as aberrantly spliced RNAs proved to be no different in this regard and were found to be implicated in various tumour hallmarks in HCC [16]. By reanalysing available transcriptomic datasets of HCC patients, we identified an upregulated circRNA hsa_circ_0062682 and evaluated its oncogenic potential. Aberrant expression of hsa_circ_0062682 has previously been implicated in lung adenocarcinoma [66] and in colorectal carcinoma where it was shown to promote tumour growth and serine metabolism [67]. By performing cell-based assays we could confirm the implication of hsa_circ_0062682 in proliferation, colony formation, migration, and invasion of HCC model cell lines. Therefore, we confirmed that upregulation of hsa_circ_0062682 could be involved in the promotion of HCC oncogenesis.

We used transcriptome analyses to further decipher the molecular mechanisms behind its oncogenic potential. Pathway enrichment analysis identified many enriched pathways of which several were in concordance with phenotypic results obtained on model cell lines (for example downregulated cell cycle, mitosis, and upregulated morphogenesis in the knocked down cell line). Additionally, pathway enrichment analysis shed a light on other perturbed pathways implicating the involvement of hsa_circ_0062682 in steroid metabolism, GPCR ligand binding, RNA splicing, and unfolded protein response. The implication of aberrant splicing [68,69], downregulated steroid metabolism [70], and upregulated unfolded protein response and ER stress [71,72] were previously associated with HCC. Moreover, enriched transcription factors in a knockdown cell model were already previously linked to the HCC pathology and can partially explain the observed phenotype. Repression of E2F1, Sp1, HIF-1α, and activation of NFκB1 in the hsa_circ_0062682 knockdown can result in a suppressive phenotype since all are associated with HCC. E2F1 is known to be an important regulator of the cell cycle and proliferation and was associated with HCC development and progression [73]. HIF-1α expression was correlated to poor prognosis of HCC patients with cirrhosis [74] and high HIF-1α and Sp1 co-expression were also found to be associated with poorer prognosis of HCC patients [75]. On the other hand, NFκB1 was found to be a suppressor of a neutrophil-driven HCC [76]. Additionally, we detected YBX1 as an enriched transcription factor in GSEA analysis for downregulated genes in a knockdown SNU-449 cell line, which further implicates its involvement. YBX1 was already described as a transcription factor, especially with the potential of regulating the expression of genes implicated in DNA repair [65].

We next focused on the interaction between hsa_circ_0062682 and YBX1 and confirmed their binding by RNA immunoprecipitation. YBX1 was also found to be upregulated in HCC and was negatively associated with the overall survival in our analysis. Other studies confirmed the proposed oncogenic effect of YBX1 in HCC [48,77,78]. YBX1 has a wide variety of functions and acts also as RBP and influences RNA stability, splicing and translation [65]. Additionally, YBX1 is already associated with the activities of transcription factors enriched in our GSEA analysis. It was associated with the activity of E2F transcription factors [79]; had a reciprocal activity with Sp1 transcription factor [80]; enhanced HIF-1α protein expression [81]; promoted NF-κB activity [82,83]; bound to TP53 transcription factor and inhibited its activity [84]; and bound to and regulated PRC2 complex activity, of which EZH2 transcription factor is a part of [85]. Furthermore, YBX1 was implicated in different signalling pathways identified as enriched in our study (TGF-beta, MAPK, PI3K-Akt, and TNF) [62,86,87,88,89,90,91]. Although hsa_circ_0062682 did not influence the localization of YBX1 to the nucleus, it can influence its role in the cytoplasmic compartment [65,92,93,94]. RNA immunoprecipitation also successfully pulled down previously described RNA partners of YBX1, also in the context of HCC, for instance, lncRNA *HULC* [58], lncRNA *AWPPH* [59], lncRNA *lincNMR* [60], and circRNA-SORE [61]. Although a competing mechanism between hsa_circ_0062682 and circRNA-SORE for binding to YBX1 was proposed, additional experiments need to be performed to confirm this hypothesis. All this data confirmed the functional interaction of hsa_circ_0062682 with YBX1 and the oncogenic potential of this interaction.

Different large-scale omics approaches have identified distinctive molecular subtypes of HCC on genomic, transcriptomic, and proteomic levels [95], indicating a very heterogeneous cancer pathology. A study investigating a panel of liver cancer cell lines identified distinct molecular subtypes [9]. Therefore, we used two distinct subclasses of HCC model cell lines, hepatoblast-like model cell lines Huh-7 and HepG2 and a mesenchymal-like model cell line SNU-449 [9]. Stable perturbations in both cell subtypes (Huh-7 and SNU-449) confirmed a promoting role of hsa_circ_0062682 in cell proliferation and colony formation. However, an opposite phenotype between the two cell lines was observed in migration, sensitivity to sorafenib, and hnRNP K cytoplasmic localization. Moreover, opposite perturbations of circRNA led to the same effect on migration within the same cell line. Such effects were described before [96] and may result from a unique combination of mutational landscape, expressed proteins, and miRNA in each of the cell lines [9]. Interestingly stable transduction of hsa_circ_0062682 also influenced the morphological changes in model cell lines, which can further indicate the involvement of this circRNA in processes such as EMT. Since circRNA-SORE plays a role in sorafenib resistance of cell lines [61] and its expression levels vary depending on the cell type, a more complex interplay between YBX1, hsa_circ_0062682 and circRNA-SORE was proposed. hnRNP K cytoplasmic localization was previously connected to the increased motility of cell lines [64] and decreased cytoplasmic levels may be correlated to the decreased motility of the SNU-449 KD cell line. Although we focused mostly on investigating the interaction between hsa_circ_0062628 and YBX1, other potential protein binding partners detected by mass spectrometry should be furthermore evaluated to unravel the functional role of the studied circRNA.

## 5. Conclusions

In summary, we confirmed that upregulation of hsa_circ_0062682 promotes HCC oncogenesis and induces systemic changes on the transcriptomic level. We identified enriched transcription factors and pathways that can explain the observed phenotype. We identified YBX1 as a protein-binding partner, along with other RNA-binding proteins. Most importantly, we uncovered a complex cell-type-specific phenotype in response to the oncogenic potential of hsa_circ_0062682. This finding is in line with different classes of HCC tumours and more studies are needed to shed a light on the molecular complexity of liver cancer.

## Data Availability

The datasets generated during the current study will be available in the Gene Expression Omnibus database (https://www.ncbi.nlm.nih.gov/geo/, accessed on 3 September 2022) under accession number GSE212472.

## Supplementary Materials

The following supporting information can be downloaded at: https://www.mdpi.com/article/10.3390/cancers14184524/s1. Figure S1: Schematic representation of the modification of a multiple cloning site in the mini-gene plasmid system pRR. Figure S2: Representative images of the wound healing assay of hsa_circ_0062682-overexpressing HepG2 cell line. Figure S3: Colony forming assay in transduced SNU-449 cell lines. Figure S4: Representative images of a wound healing assay of overexpressing Huh-7 (A) and in a knockdown of Huh-7 (B). Figure S5: Representative images of a wound healing assay of overexpressing SNU-449 (A) and in a knockdown of SNU-449 (B). Figure S6: RT-qPCR validation of differentially expressed genes identified by microarray analysis in the knockdown cell line SNU-449. Figure S7: RT-qPCR validation of differentially expressed genes identified by microarray analysis in the transient overexpression of hsa_circ_0062682 in Huh-7 cell line. Figure S8: GSEA analysis of upregulated (red colour) and downregulated (blue colour) pathways of Huh-7 transient overexpression. Figure S9: Volcano plots from Enrichr analysis of enriched gene sets in TRRUST transcription factors of upregulated (left) and downregulated (right) differentially expressed genes in SNU-449 knockdown cell line. Figure S10: Enrichment of RNA species in biotinylated oligonucleotide pulldowns normalized the input. Figure S11: Viability of sorafenib treated overexpression cell lines measured by colorimetric CCK-8 assay. Figure S12: Relative expression of RNA species in human HCC model cell lines (Huh-7, HepG2, SNU-449) and human hepatic stellate cell line LX-2, normalized to the immortalized human hepatocyte cell line THLE5B. Figure S13: YBX1 localization in hsa_circ_0062682 stable cell lines identified by Western blotting. The datasets generated during the current study are available in the Gene Expression Omnibus database (https://www.ncbi.nlm.nih.gov/geo/) under accession number GSE 212472. Table S1: Primers, siRNA sequences and biotinylated oligonucleotide sequences used in the experiments. Table S2: Upregulated and downregulated circRNA in HCC, identified in GSE94508 and GSE97332. Table S3: Transcriptome analysis of transient overexpression of hsa_circ_0062682 in Huh-7 cell line and stabled knockdown of hsa_circ_0062682 in SNU-449 cell line. Table S4: Pathway enrichment analysis of transcriptome data from knockdown of hsa_circ_0062682 in SNU-449 and overexpression in Huh-7 cell line as described in Reimand et al. 2019. Table S5: Enrichr analysis of enriched pathways and transcription factors of differentially expressed genes in SNU-449 knockdown. Table S6: Enrichr analysis of enriched pathways of differentially expressed genes separately for upregulated and downregulated genes in SNU-449 knockdown. Table S7: Enrichr analysis of enriched transcription factors of differentially expressed genes separately for upregulated and downregulated genes in SNU-449 knockdown. Table S8: Potential binding sites of RBPs in hsa_circ_0062682 identified in silico. Table S9: Proteomics analysis of biotinylated oligonucleotide pulldown experiment. Table S10: Enriched Reactome and KEGG pathways in STRING analysis of proteins identified by proteomics analysis. Supplementary data: Uncropped images of whole western blots.
