# Supplementary material for: Circular RNA hsa_circ_0062682 Binds to YBX1 and Promotes Oncogenesis in Hepatocellular Carcinoma
Source: Cancers (Basel). 2022 Sep 19;14(18):4524. doi: 10.3390/cancers14184524 (PMC9497178; doi:10.3390/cancers14184524)
Supplement: Supplementary file 1 [file cancers-14-04524-s001.zip › cancers-1865575-supplementary data.pdf]

Figure 6E – YBX1, Huh-7 OE

Upper membrane, from right to left (input, antisense, sense, anti-eGFP)

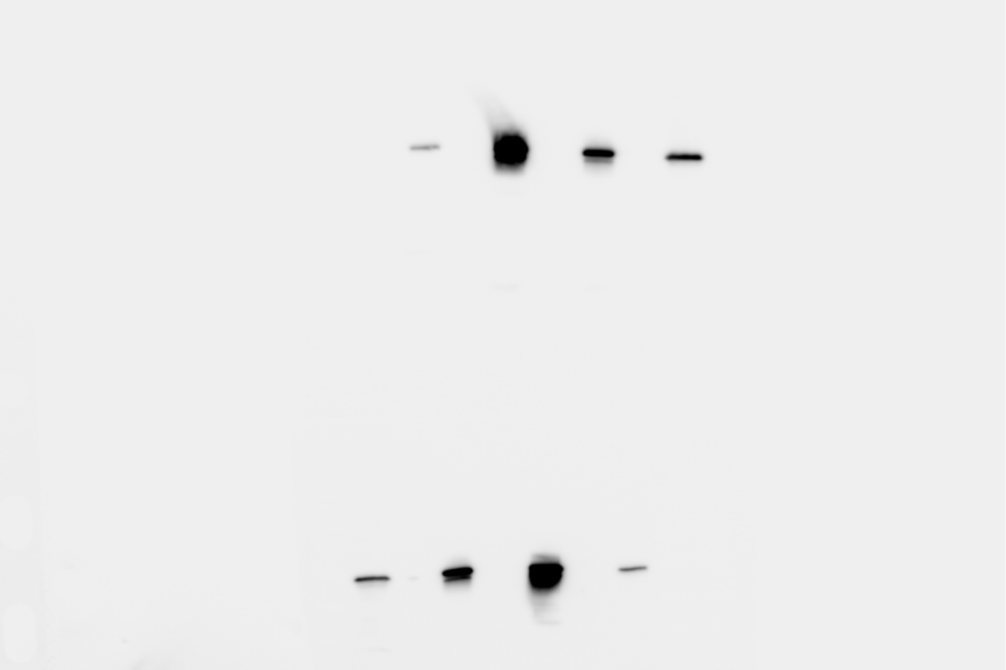

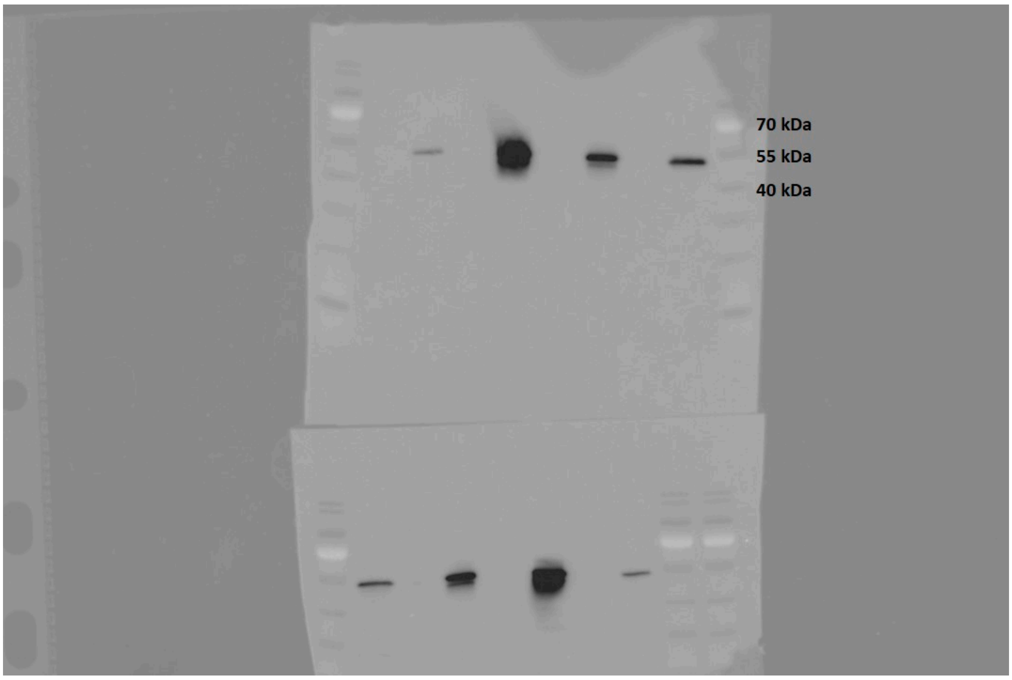

Figure 6E – YBX1, SNU-449 OE

Lower membrane, from left to right (input, antisense, sense, anti-eGFP)

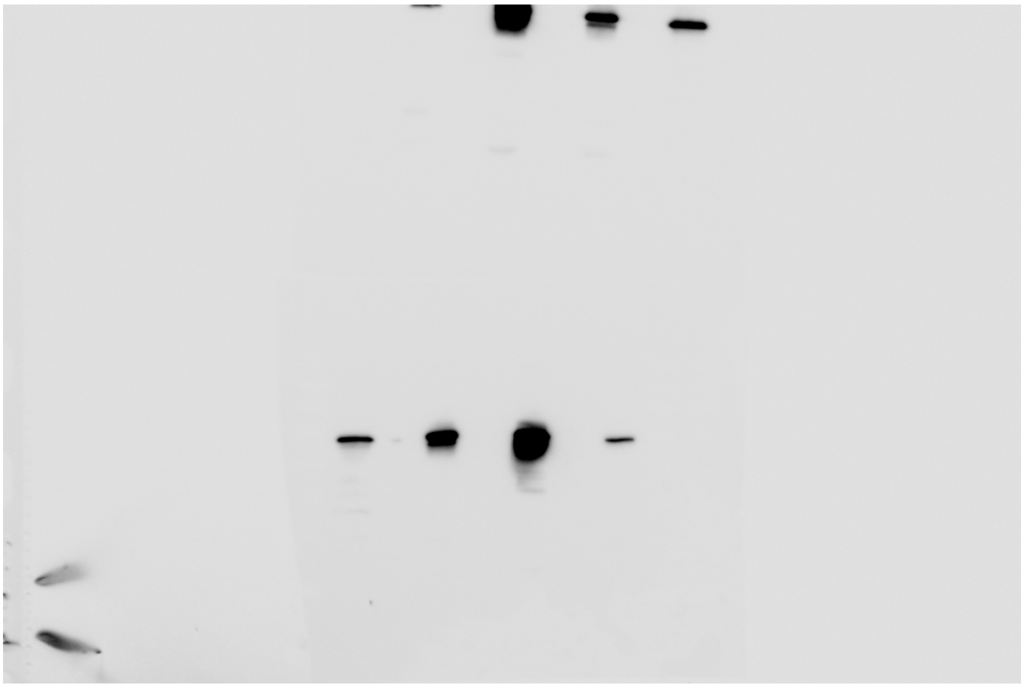

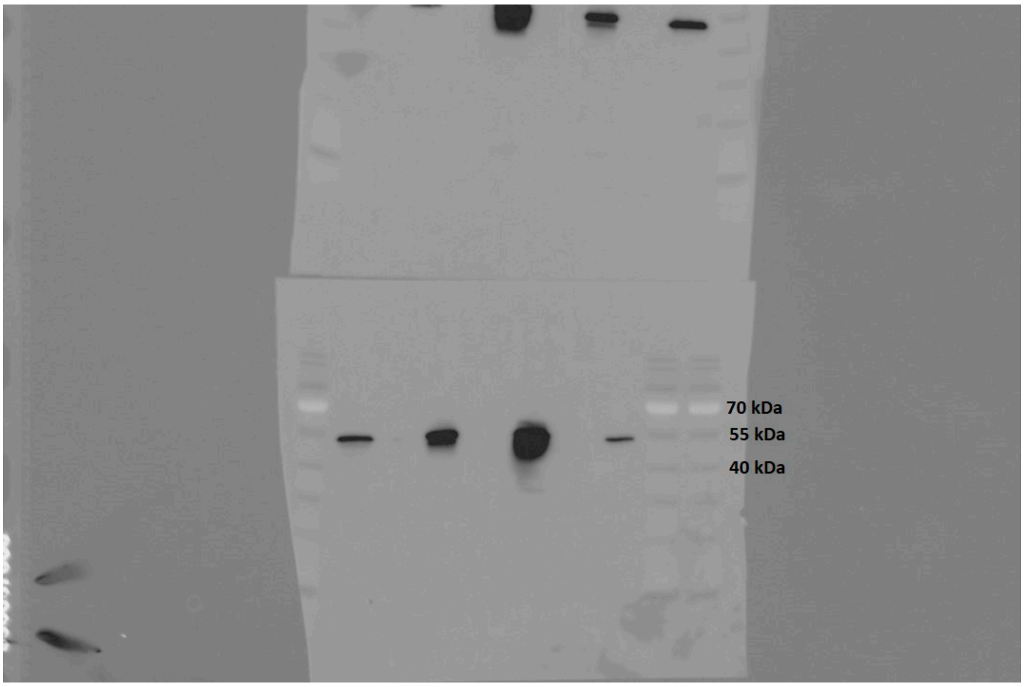

Figure 6E – IGF2BP1, Huh-7 OE

Upper membrane, from right to left (input, antisense, sense, anti-eGFP)

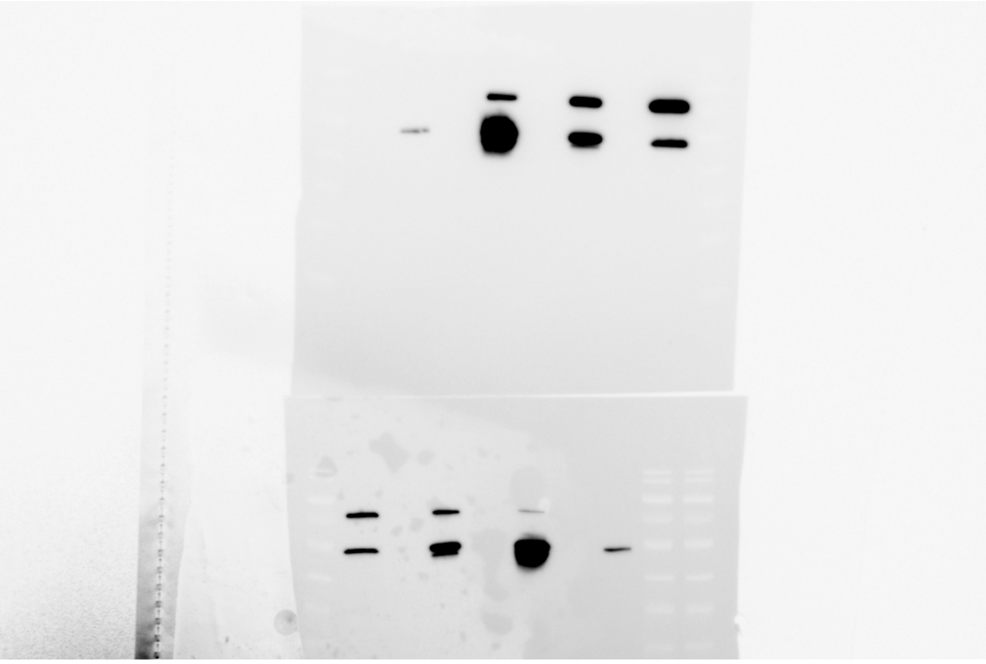

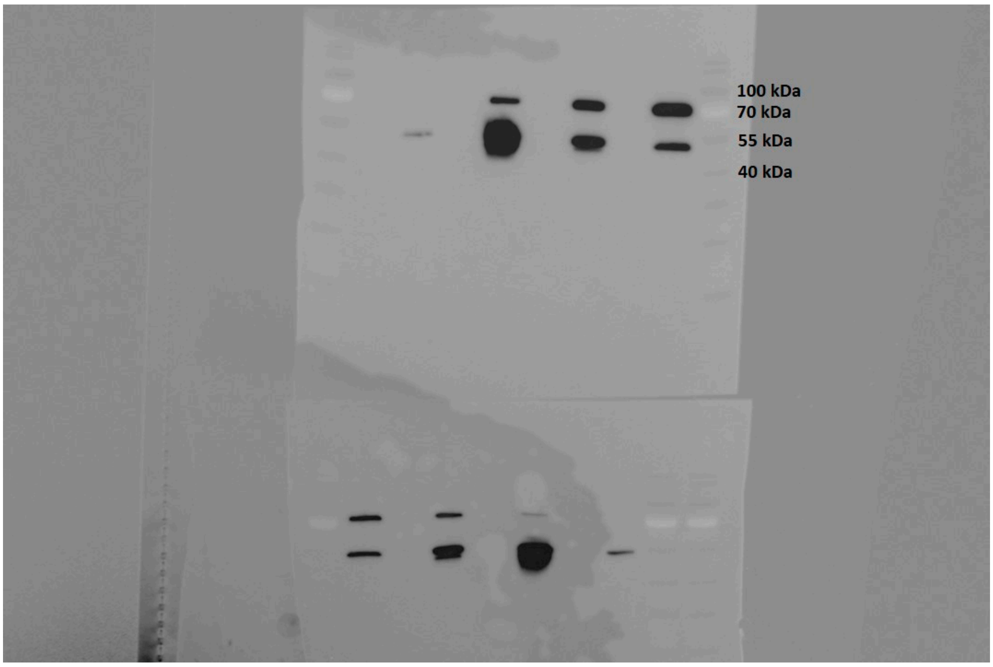

Figure 6E – IGF2BP1, SNU-449 OE

Lower membrane, from left to right (input, antisense, sense, anti-eGFP)

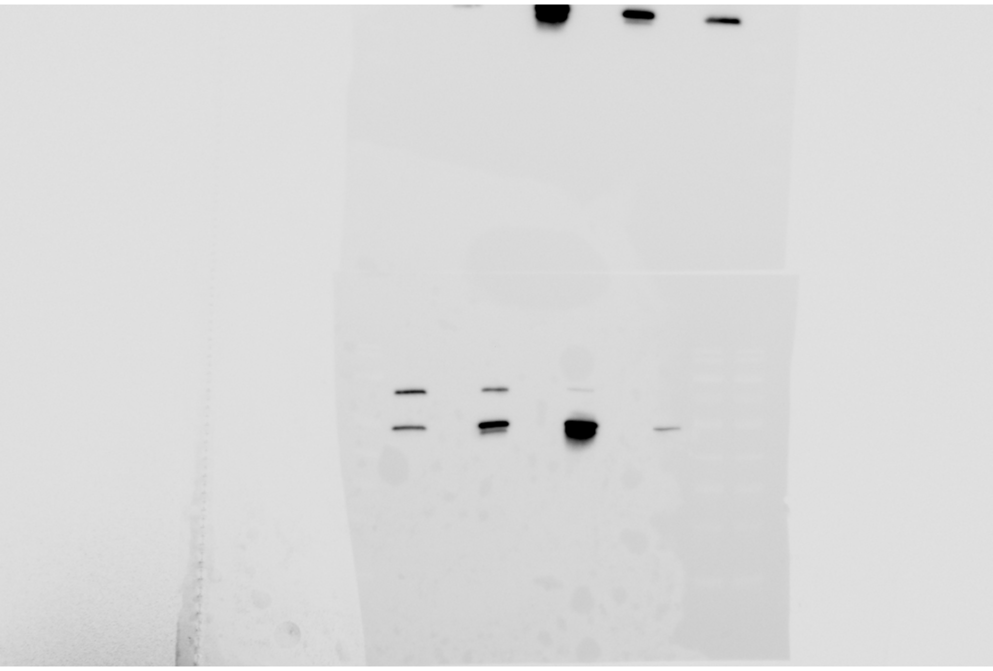

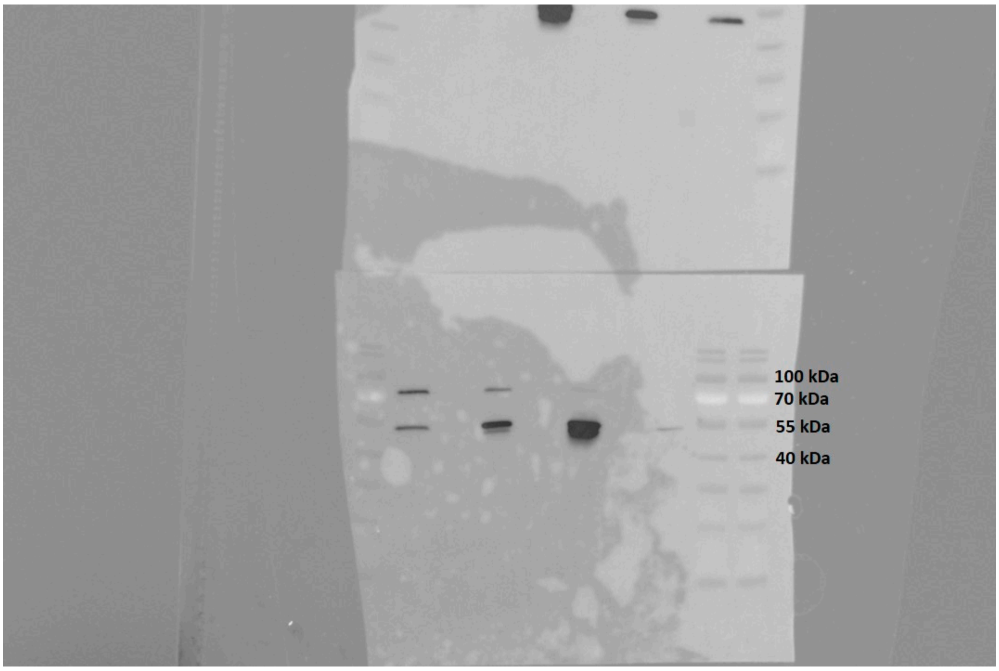

Figure 6E – HNRNPC1/2

Huh-7 OE, upper membrane, from right to left (input, antisense, sense, anti-eGFP)

SNU-449 OE, lower membrane, from left to right (input, antisense, sense, anti-eGFP)

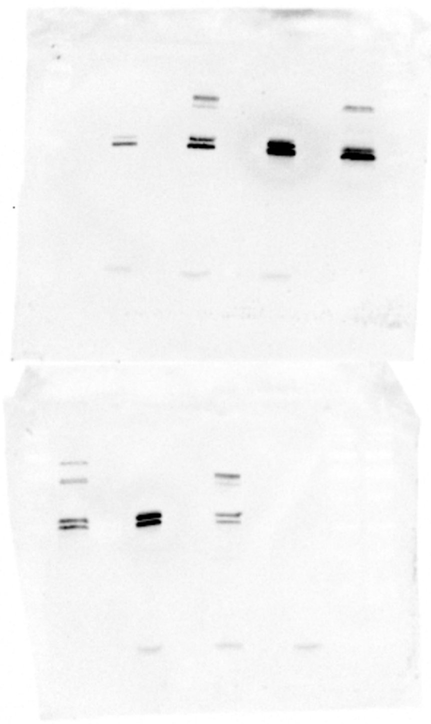

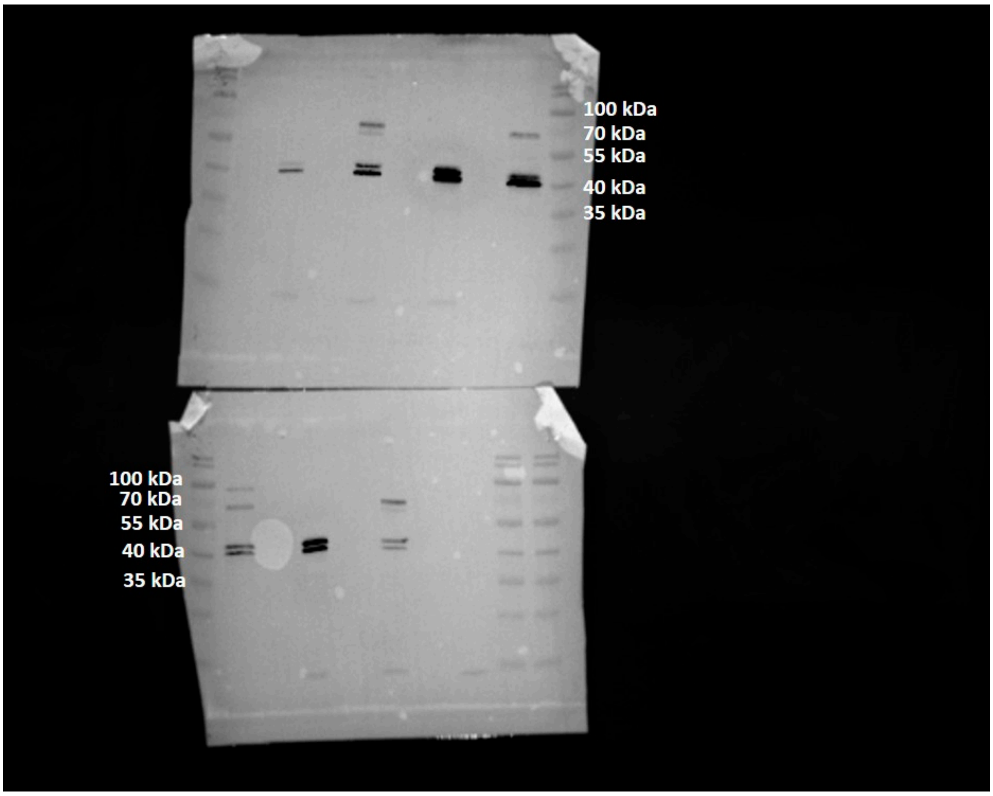

Figure 6E – HNRNPK

Huh-7 OE, upper membrane, from right to left (input, antisense, sense, anti-eGFP)

SNU-449 OE, lower membrane, from left to right (input, antisense, sense, anti-eGFP)

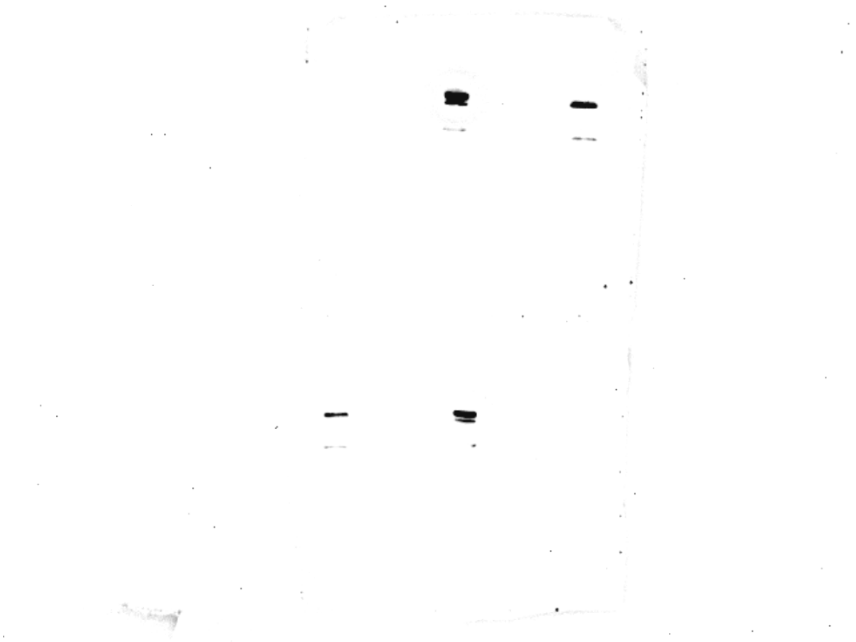

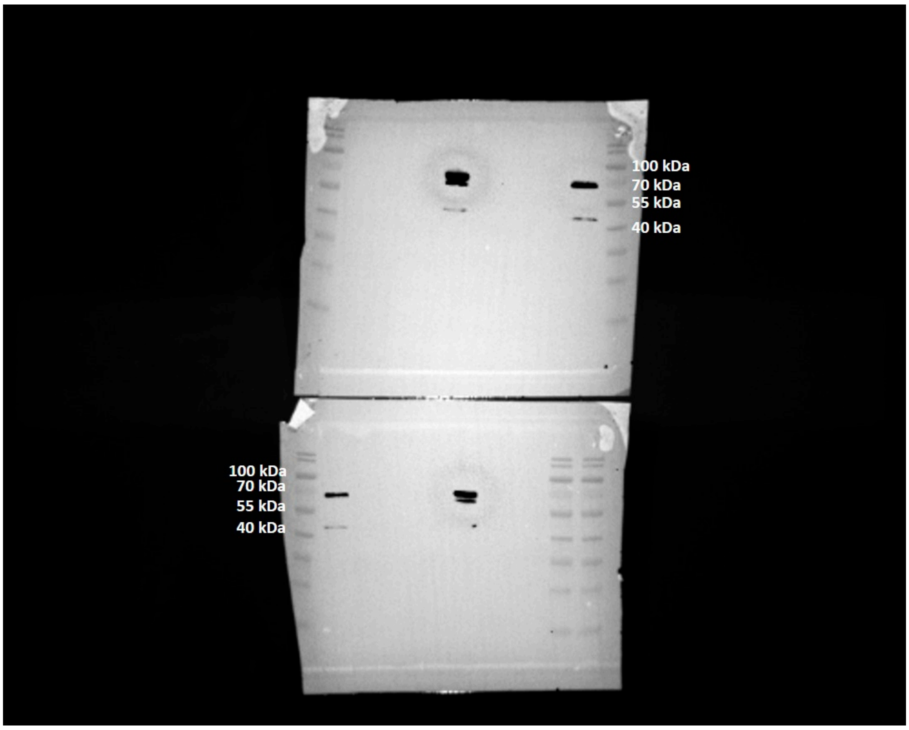

Figure 6E – CSNK2A1

Huh-7 OE, upper membrane, from right to left (input, antisense, sense, anti-eGFP)

SNU-449 OE, lower membrane, from left to right (input, antisense, sense, anti-eGFP)

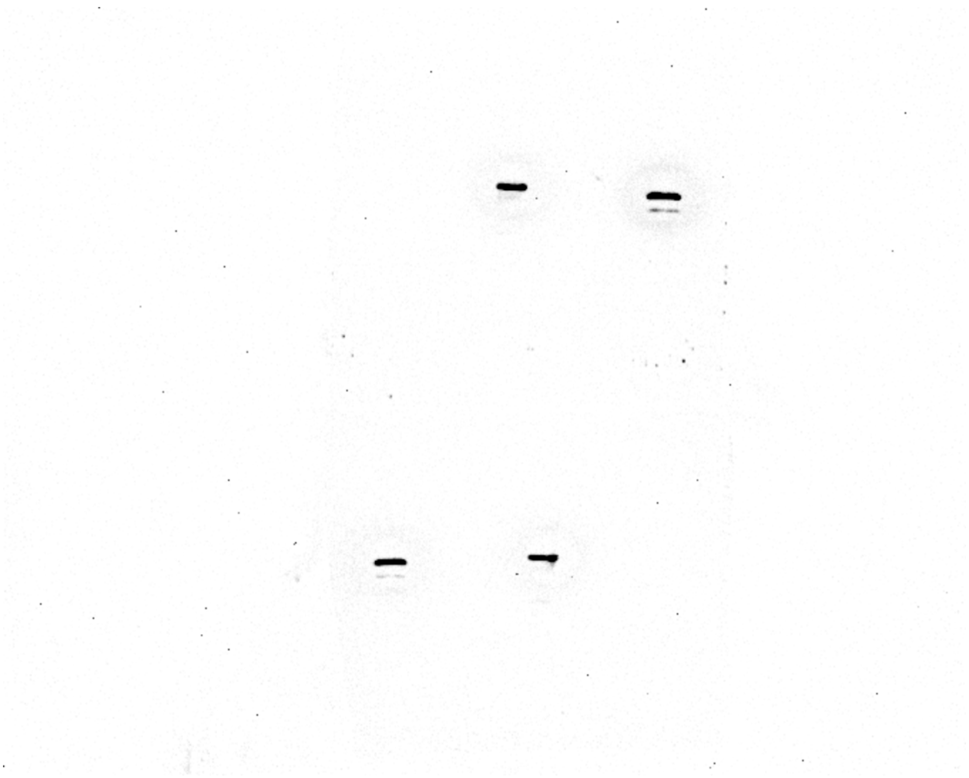

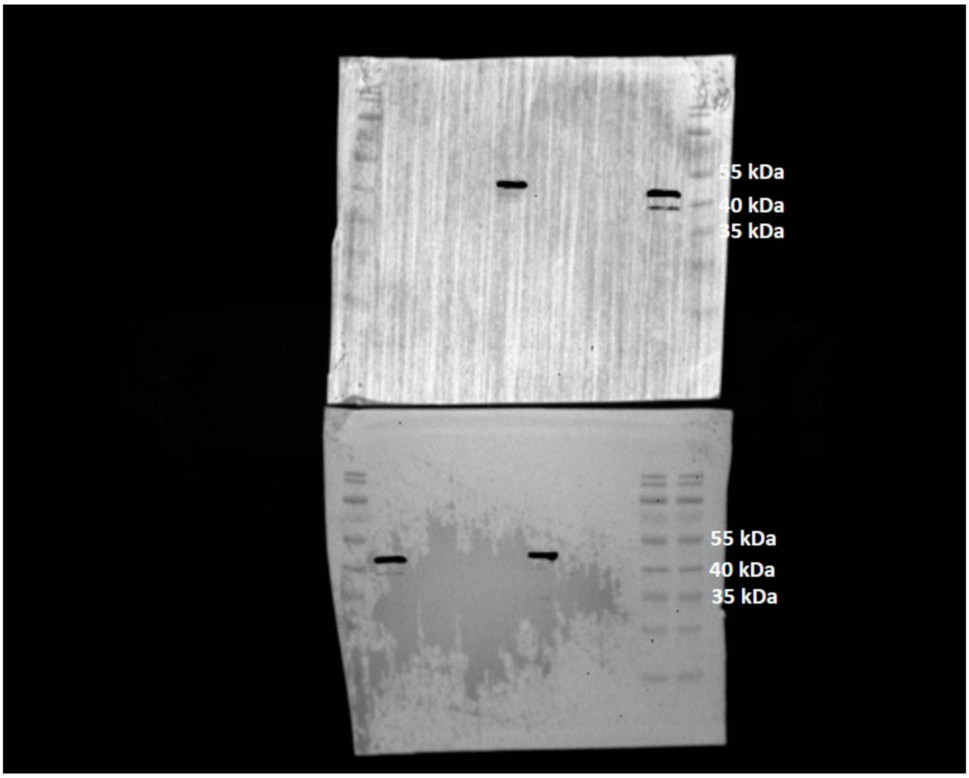

Figure 6E – ENO1

Huh-7 OE, upper membrane, from right to left (input, antisense, sense, anti-eGFP)

SNU-449 OE, lower membrane, from left to right (input, antisense, sense, anti-eGFP)

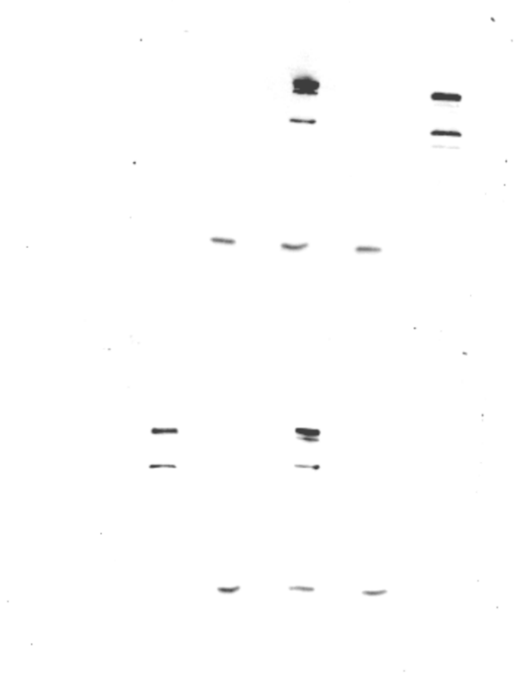

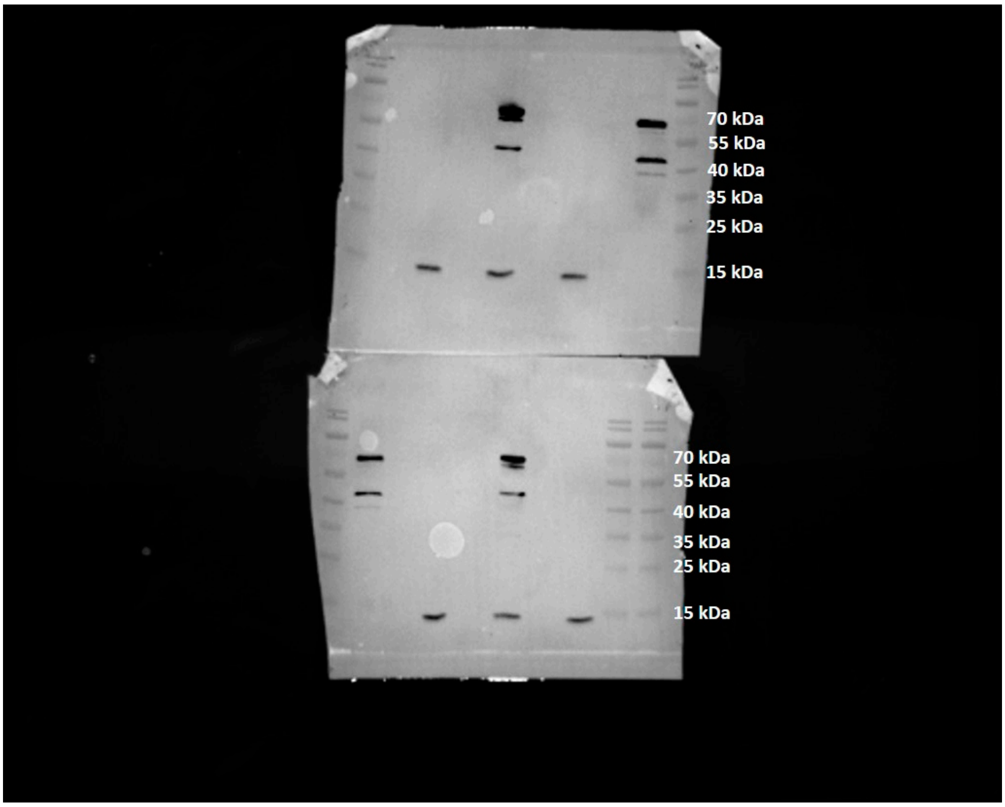

Figure 7A

From left to right (input, anti-YBX1, anti-IgG, anti-YBX1, anti-IgG, anti-YBX1, anti-IgG)

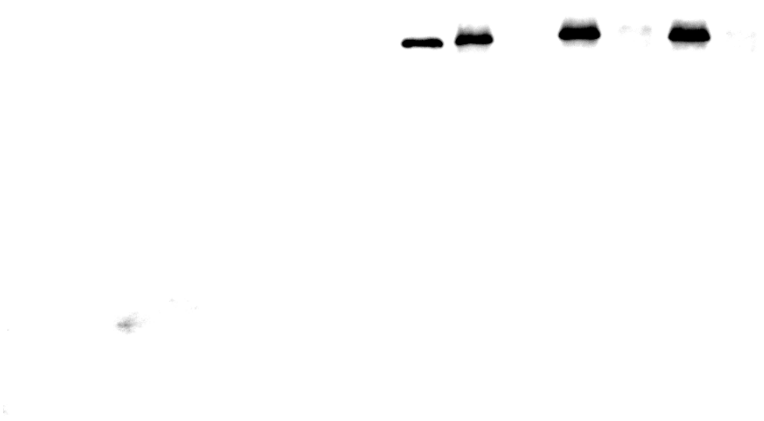

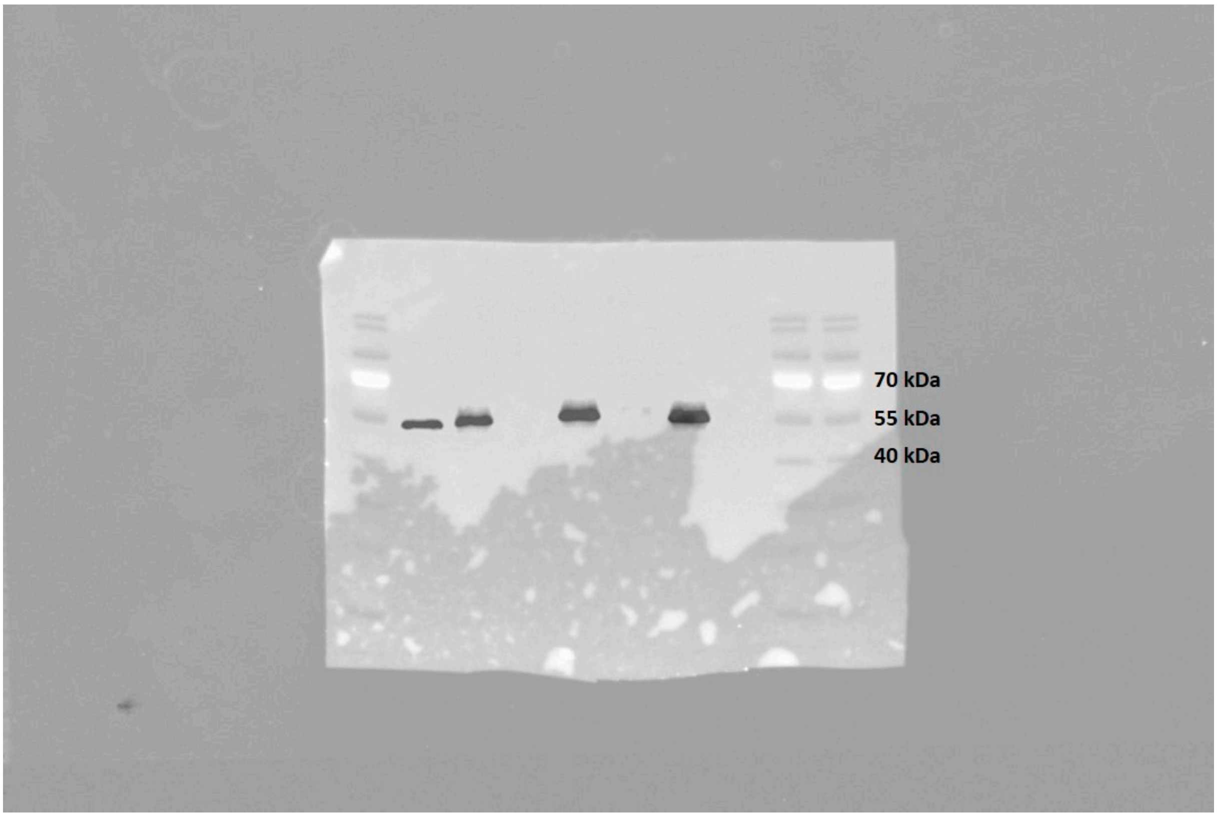

Figure 7E

HNRNPK localization, SNU-449

From left to right

Top – (scramble (cytoplasm, nucleus); KD(cytoplasm, nucleus), scramble (cytoplasm, nucleus), KD (cytoplasm, nucleus))

Bottom left – (control OE (cytoplasm, nucleus); OE (cytoplasm, nucleus); control OE (cytoplasm, nucleus); OE (cytoplasm, nucleus))

Bottom right – (control OE (cytoplasm, nucleus); OE (cytoplasm, nucleus); scramble (cytoplasm, nucleus); KD (cytoplasm, nucleus))

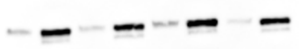

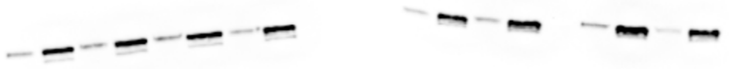

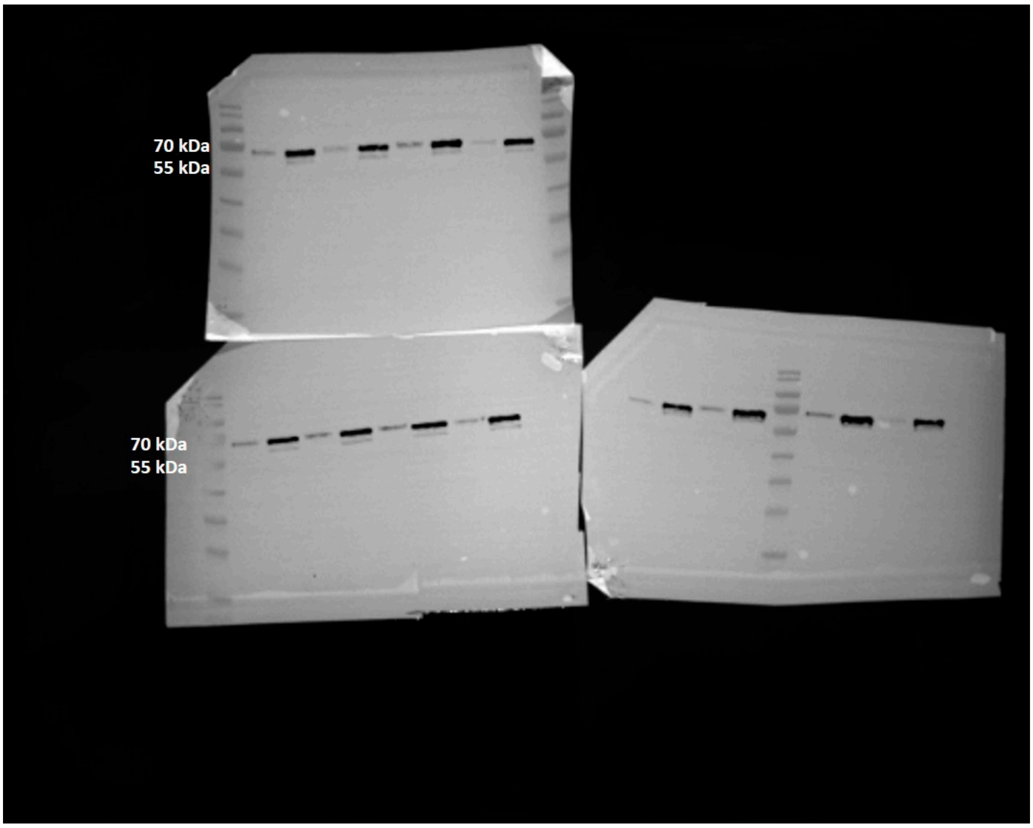
70 kDa
55 kDa
70 kDa
55 kDa

Figure 7E

Lamin A/C localization, SNU-449

From left to right

Top – (scramble (cytoplasm, nucleus); KD(cytoplasm, nucleus), scramble (cytoplasm, nucleus), KD (cytoplasm, nucleus))

Bottom left – (control OE (cytoplasm, nucleus); OE (cytoplasm, nucleus); control OE (cytoplasm, nucleus); OE (cytoplasm, nucleus))

Bottom right – (control OE (cytoplasm, nucleus); OE (cytoplasm, nucleus); scramble (cytoplasm, nucleus); KD (cytoplasm, nucleus))

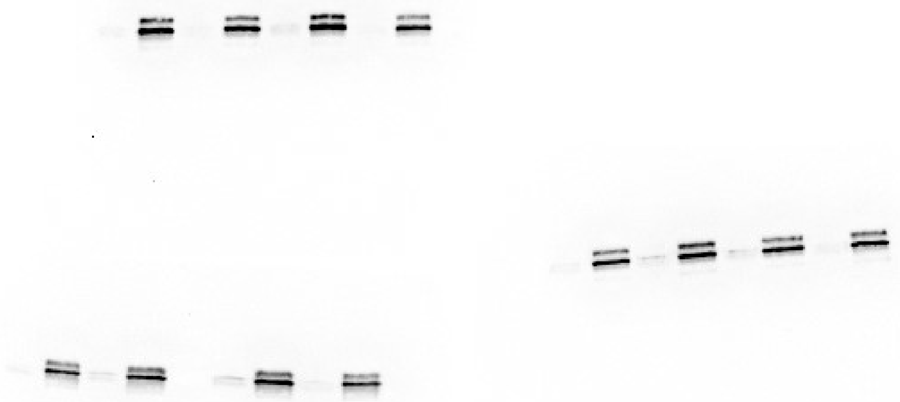

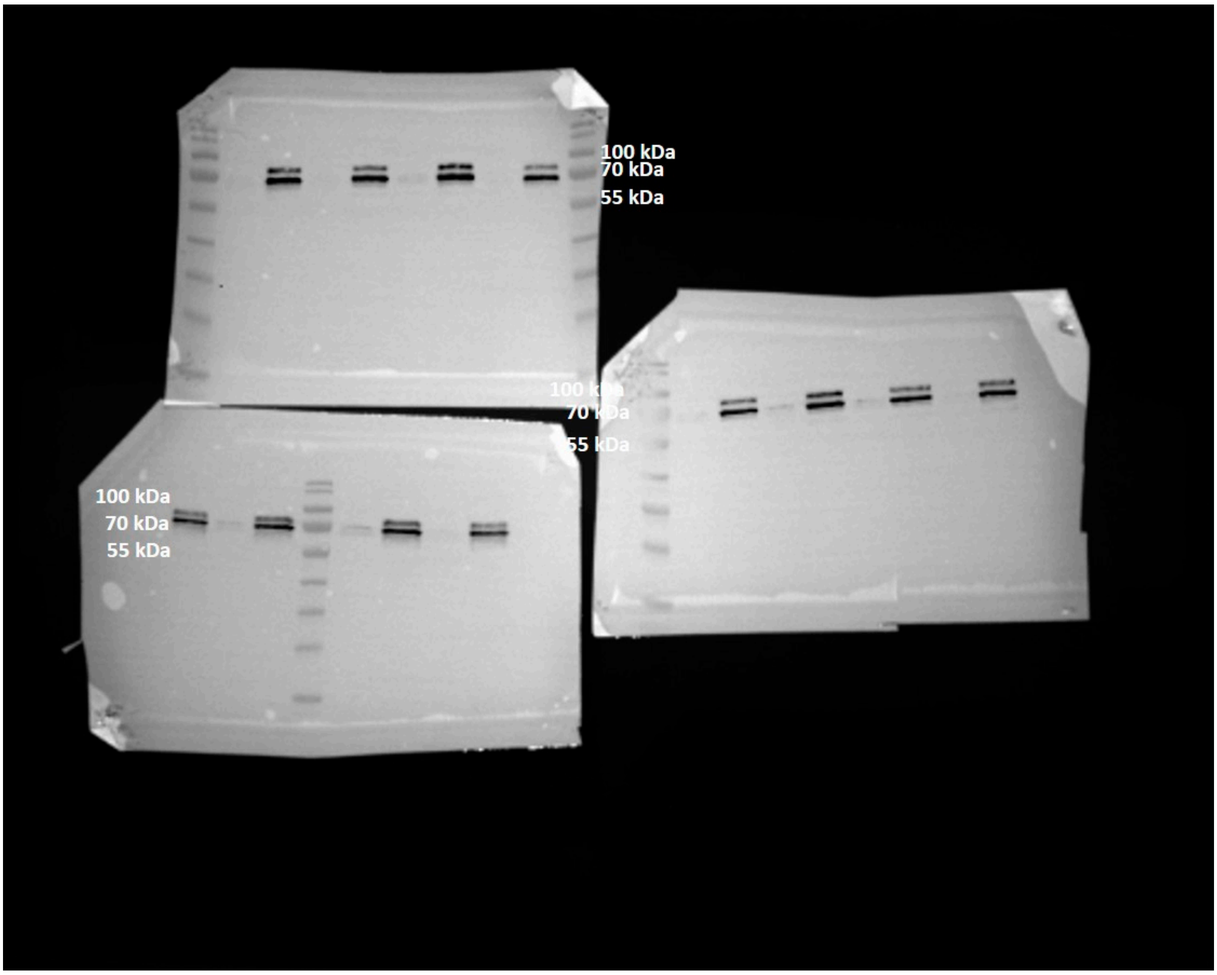
100 kDa
70 kDa
55 kDa
100 kDa
70 kDa
55 kDa
100 kDa
70 kDa
55 kDa

Figure 7E

HNRNPK localization, Huh-7

From left to right

Top – (KD (nucleus, cytoplasm); scramble (nucleus, cytoplasm); KD (nucleus, cytoplasm); scramble (nucleus, cytoplasm))

Bottom left – (control OE (cytoplasm, nucleus); OE (cytoplasm, nucleus); control OE (cytoplasm, nucleus); OE (cytoplasm, nucleus))

Bottom right – (control OE (cytoplasm, nucleus); OE (cytoplasm, nucleus); scramble (cytoplasm, nucleus); KD (cytoplasm, nucleus))

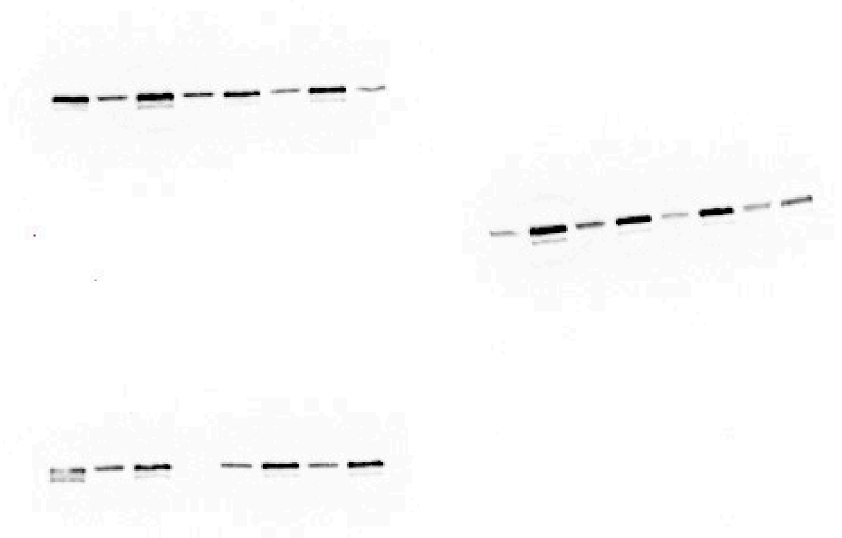

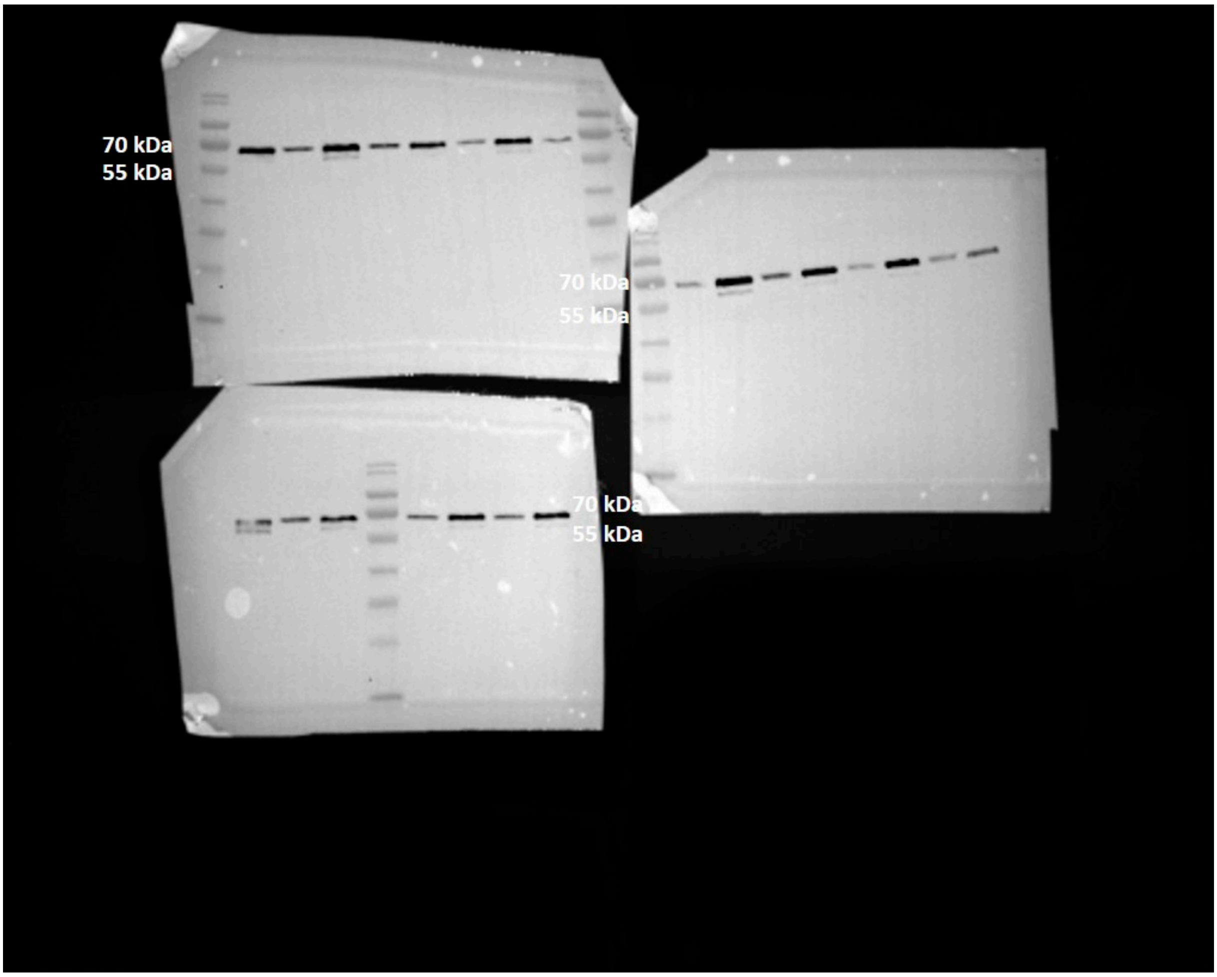
70 kDa
55 kDa
70 kDa
55 kDa
70 kDa
55 kDa

Figure 7E

Lamin A/C localization, Huh-7

From left to right

Top – (KD (nucleus, cytoplasm); scramble (nucleus, cytoplasm); KD (nucleus, cytoplasm); scramble (nucleus, cytoplasm))

Bottom left – (control OE (cytoplasm, nucleus); OE (cytoplasm, nucleus); control OE (cytoplasm, nucleus); OE (cytoplasm, nucleus))

Bottom right – (control OE (cytoplasm, nucleus); OE (cytoplasm, nucleus); scramble (cytoplasm, nucleus); KD (cytoplasm, nucleus))

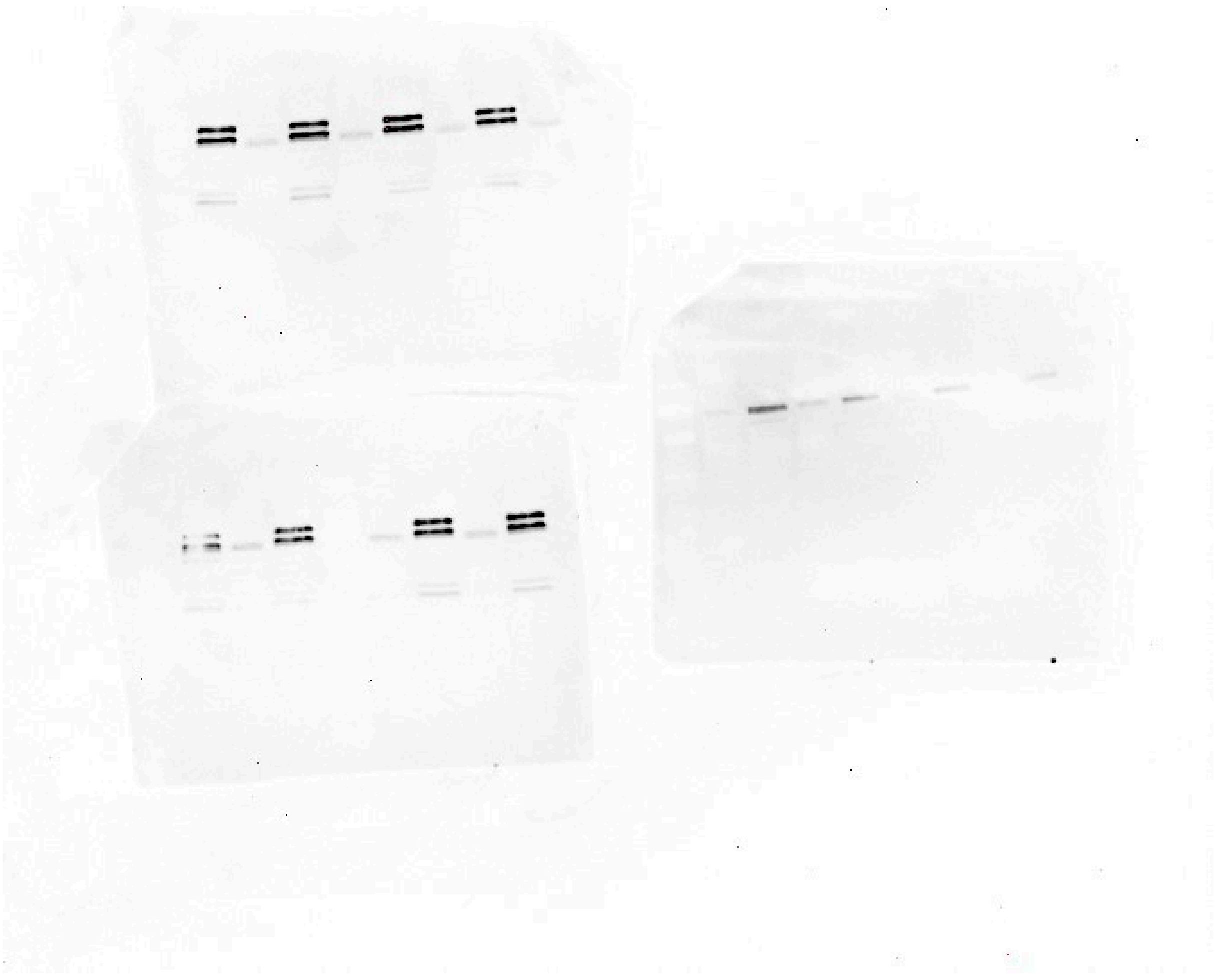

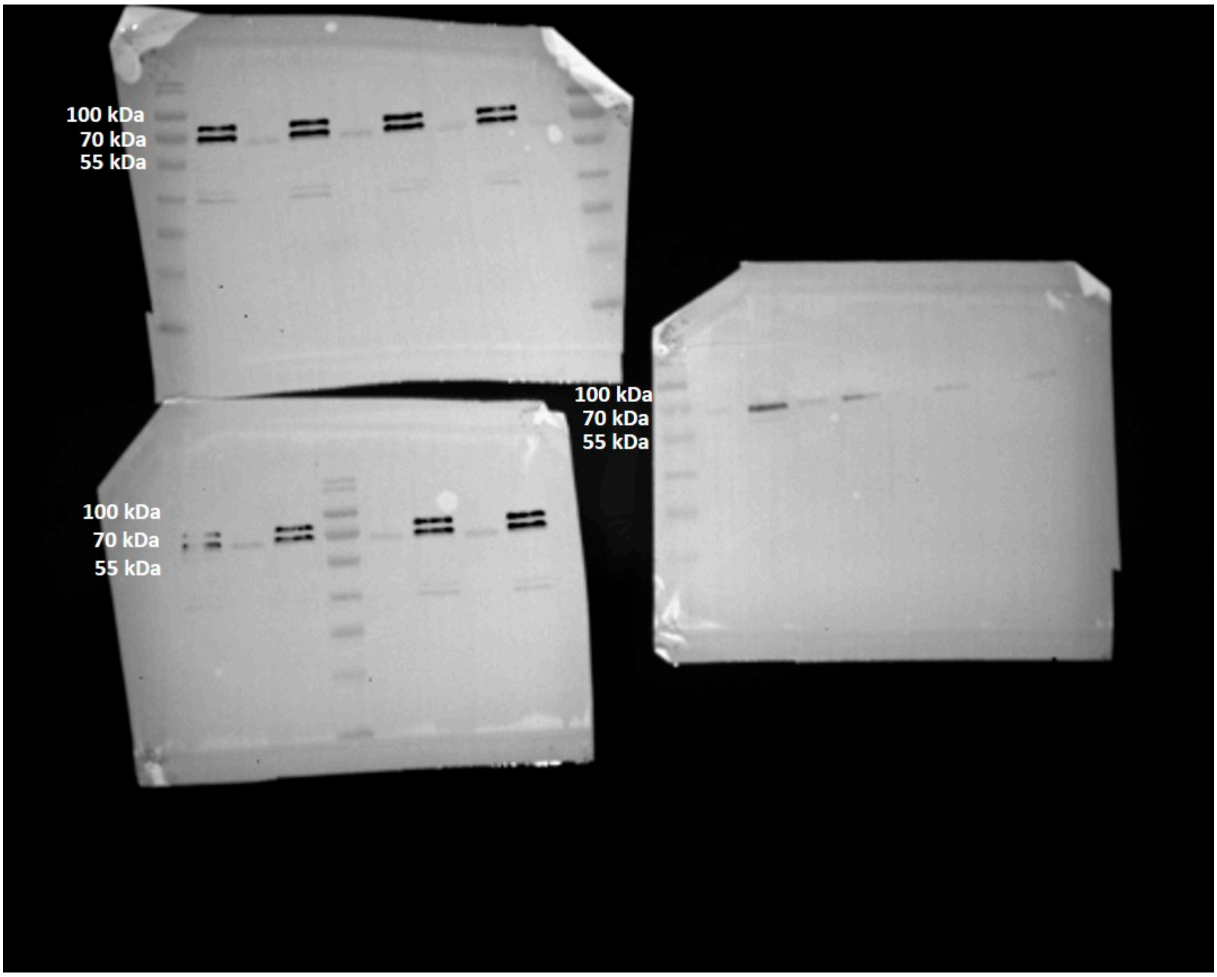
100 kDa
70 kDa
55 kDa
100 kDa
70 kDa
55 kDa
100 kDa
70 kDa
55 kDa
