# Supplementary material for: Circular RNA hsa_circ_0062682 Binds to YBX1 and Promotes Oncogenesis in Hepatocellular Carcinoma
Source: Cancers (Basel). 2022 Sep 19;14(18):4524. doi: 10.3390/cancers14184524 (PMC9497178; doi:10.3390/cancers14184524)
Supplement: Supplementary file 1 [file cancers-14-04524-s001.zip › cancers-1865575-Supplementary-materials-sec_revision.pdf]

Supplementary Materials

Circular RNA hsa_circ_0062682 binds to YBX1 and promotes oncogenesis in hepatocellular carcinoma

Rok Razpotnik [1], Robert Vidmar[2], Marko Fonović[2], Damjana Rozman[1] and Tadeja Režen [1,*]

[1] Centre for Functional Genomics and Bio-Chips, Institute of Biochemistry and Molecular Genetics, Faculty of Medicine, University of Ljubljana, 1000 Ljubljana, Slovenia

[2] Department of Biochemistry and Molecular and Structural Biology, Jožef Stefan Institute, 1000 Ljubljana, Slovenia

* Correspondence: tadeja.rezen@mf.uni-lj.si; Tel.: +386-1543-7592

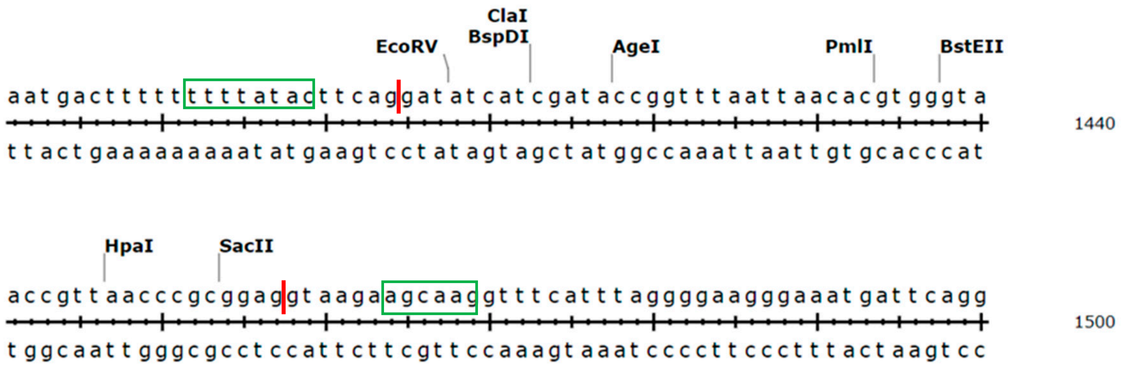

Figure S1: Schematic representation of the modification of a multiple cloning site in the mini-gene plasmid system pRR. Green colour indicates sites of introduced mutations. Red colour indicates splice sites.

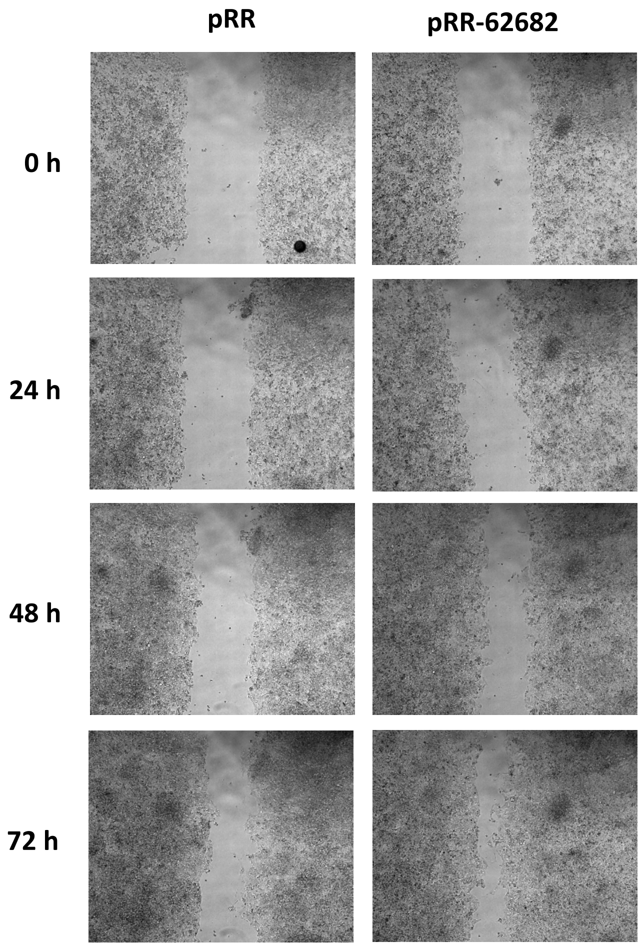

Figure S2: Representative images of the wound healing assay of hsa_circ_0062682-overexpressing HepG2 cell line; pRR – empty plasmid, pRR-62682 – plasmid for hsa_circ_0062682 overexpression.

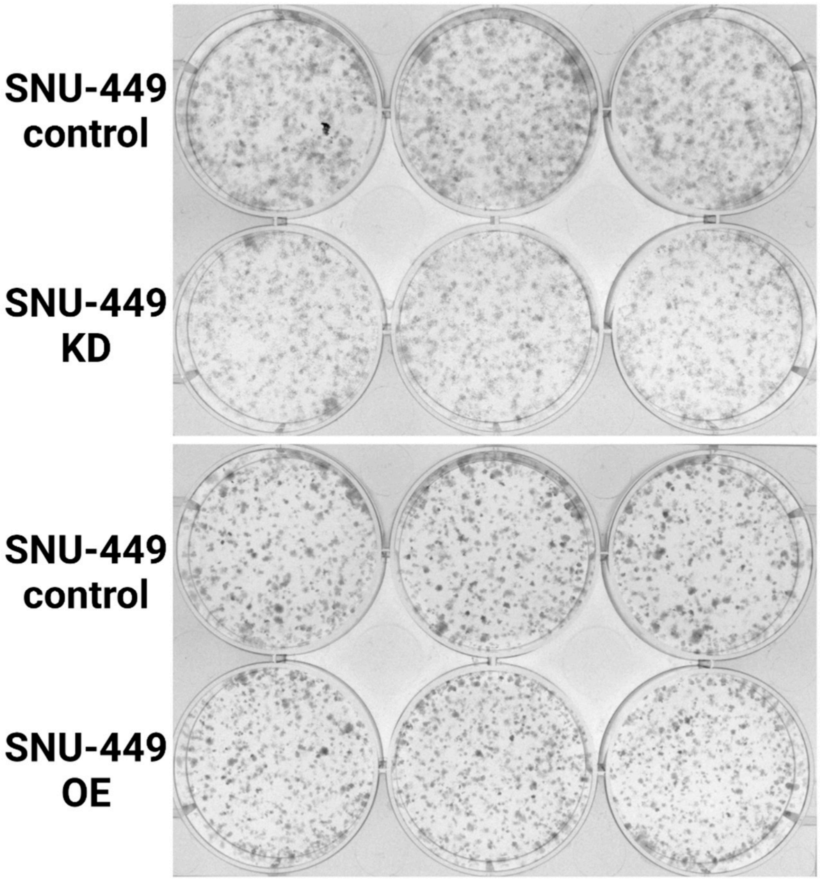

Figure S3: Colony forming assay in transduced SNU-449 cell lines; KD – knockdown (2.000 cells) ; OE – overexpression (1.000 cells).

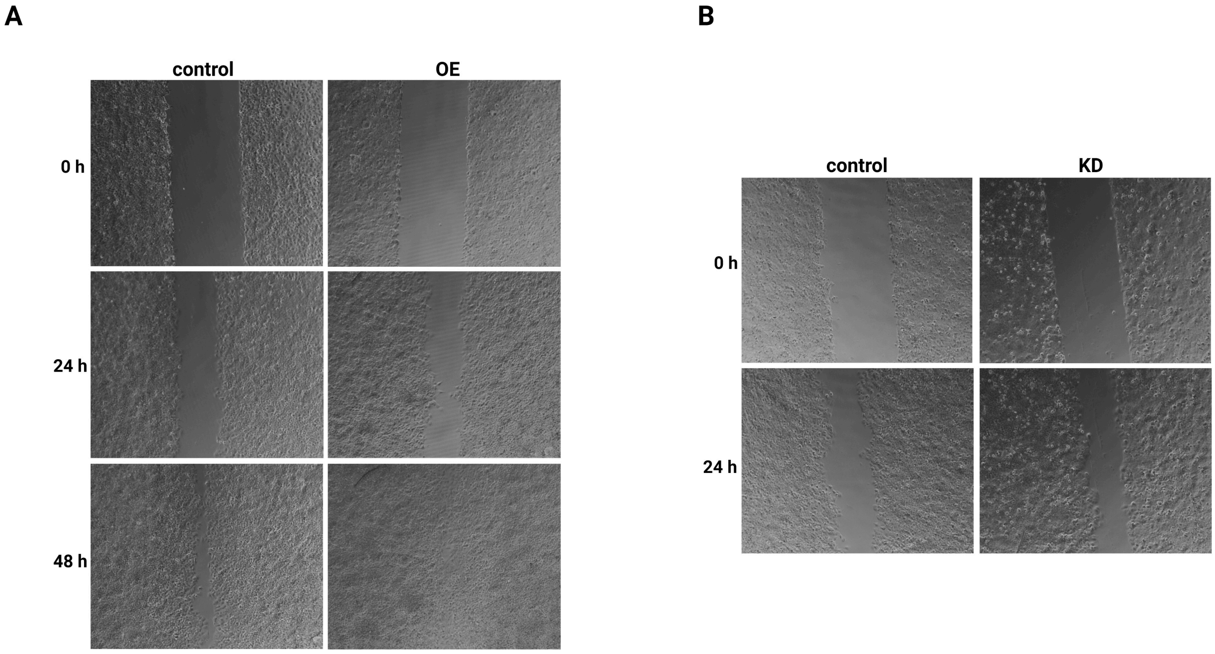

Figure S4: Representative images of a wound healing assay of overexpressing Huh-7 (A) and in a knockdown of Huh-7 (B); OE – overexpression, KD – knockdown.

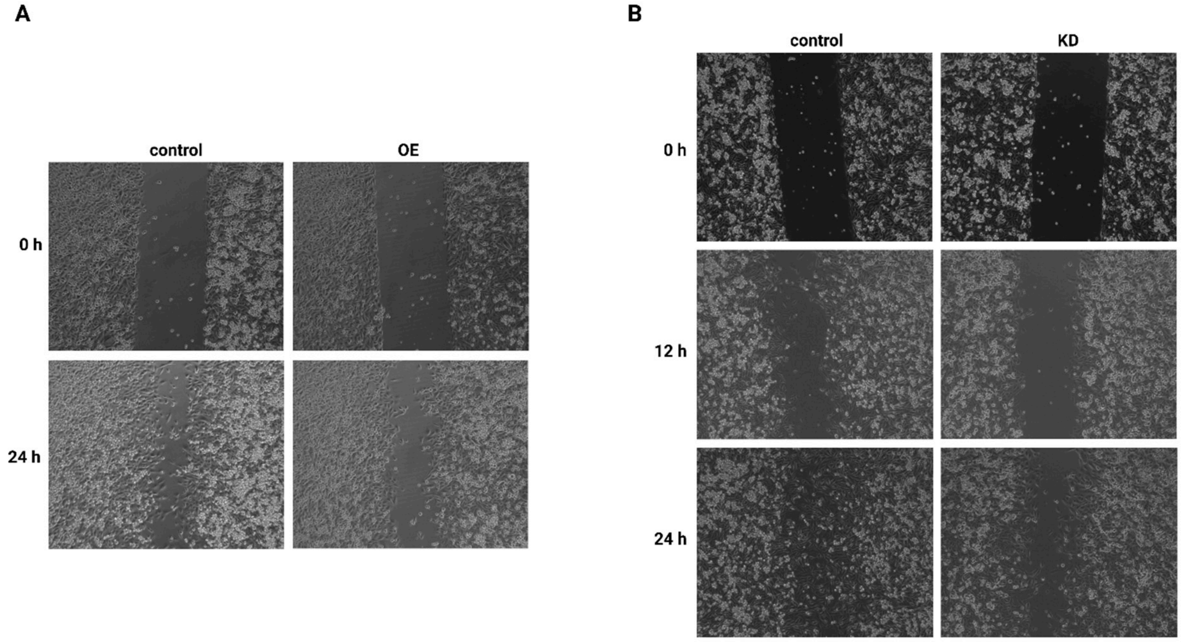

Figure S5: Representative images of a wound healing assay of overexpressing SNU-449 (A) and in a knockdown of SNU-449 (B); OE – overexpression, KD – knockdown.

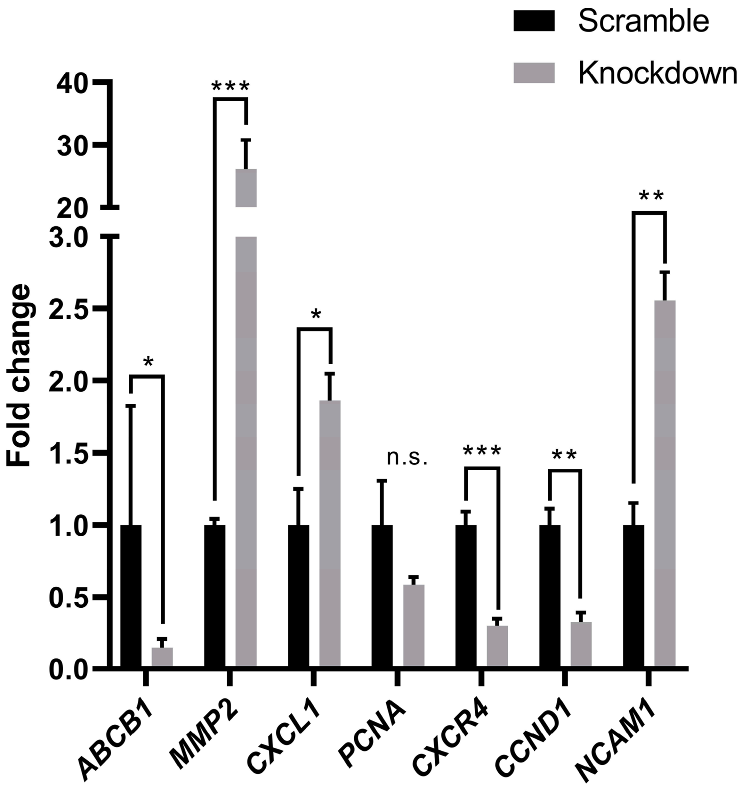

Figure S6: RT-qPCR validation of differentially expressed genes identified by microarray analysis in the knockdown cell line SNU-449. We analyzed the expression of target candidates of transcription factors E2F1

(*CCND1, PCNA*), NFKB1 (*MMP2, CXCL1, NCAM1*) and HIF-1A (*ABCB1, CXCR4*). Results are represented as averages and error bars represent standard deviation. * p-value ≤ 0.05; ** p-value ≤ 0.01; *** p-value ≤ 0.001; n.s. – not significant, Student`s t-test.

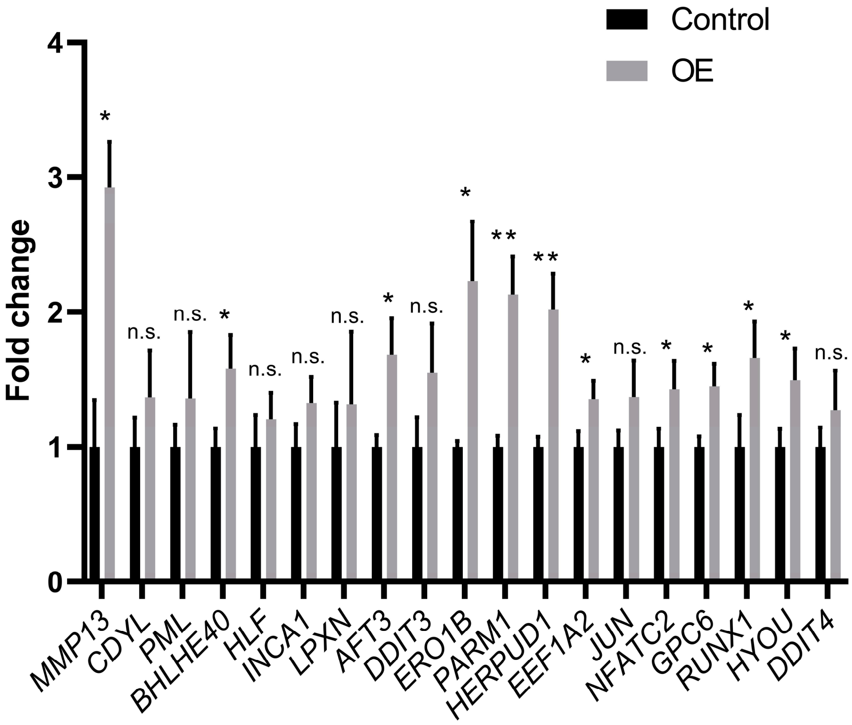

Figure S7: RT-qPCR validation of differentially expressed genes identified by microarray analysis in the transient overexpression of hsa_circ_0062682 in Huh-7 cell line. Results are represented as averages and error bars represent standard deviation. * p-value ≤ 0.05; ** p-value ≤ 0.01; n.s. – not significant, Student`s t-test.

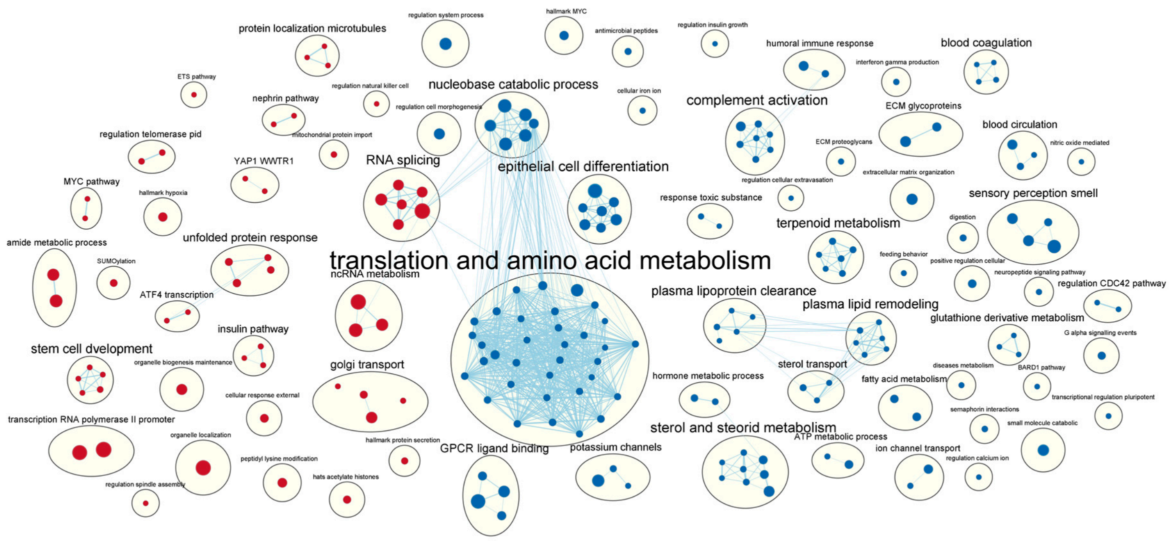

Figure S8: GSEA analysis of upregulated (red colour) and downregulated (blue colour) pathways of Huh-7 transient overexpression

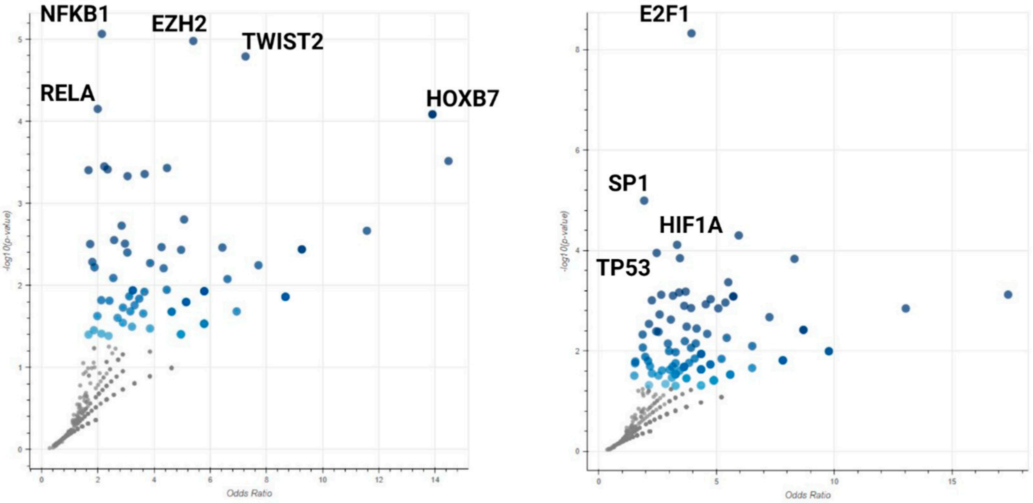

Figure S9: Volcano plots from Enrichr analysis of enriched gene sets in TRRUST transcription factors of upregulated (left) and downregulated (right) differentially expressed genes in SNU-449 knockdown celll line.

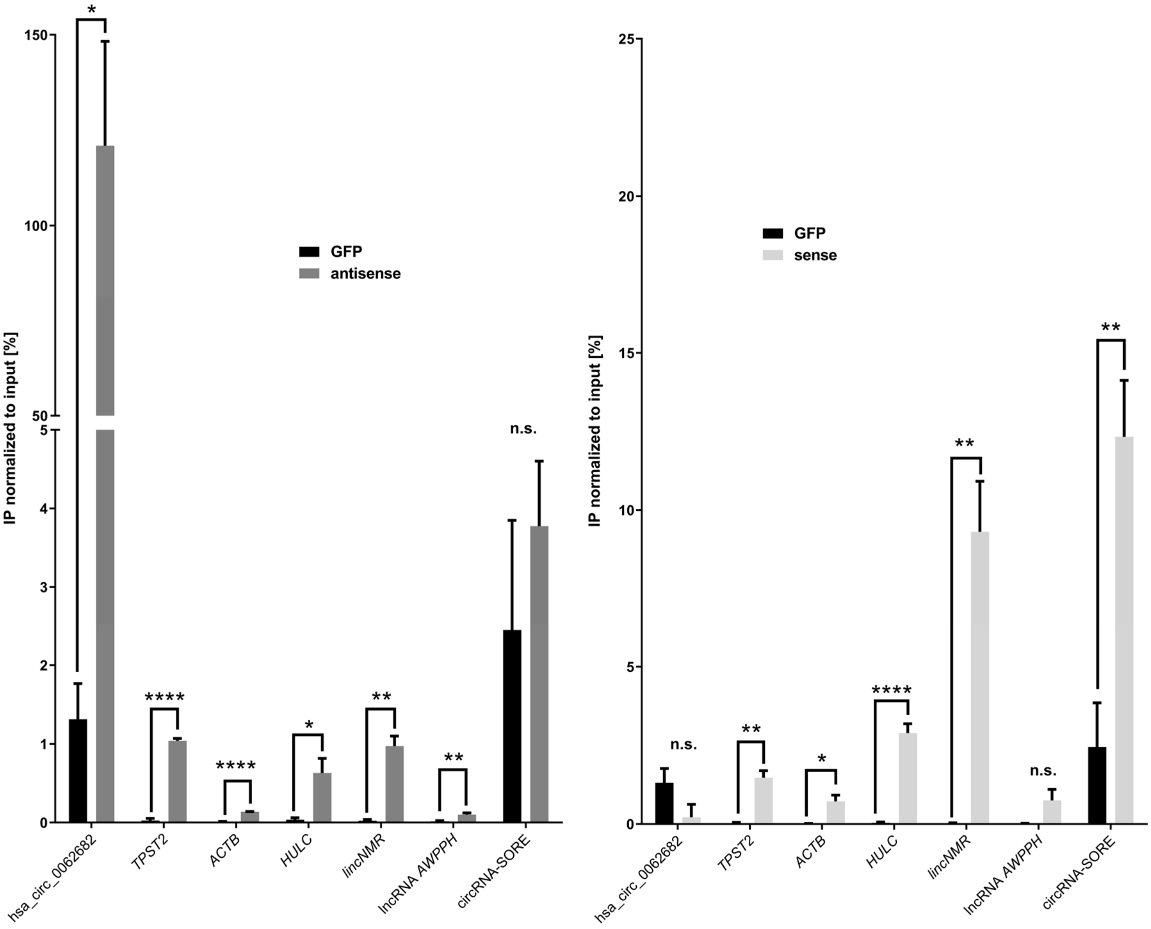

Figure S10: Enrichment of RNA species in biotinylated oligonucleotide pulldowns normalized the input; antisense (left), sense (right) (n = 3). Results are represented as averages and error bars represent standard deviation. * p-value ≤ 0.05; ** p-value ≤ 0.01; *** p-value ≤ 0.001; **** p-value ≤ 0.0001; n.s. – not significant, Student`s t-test.

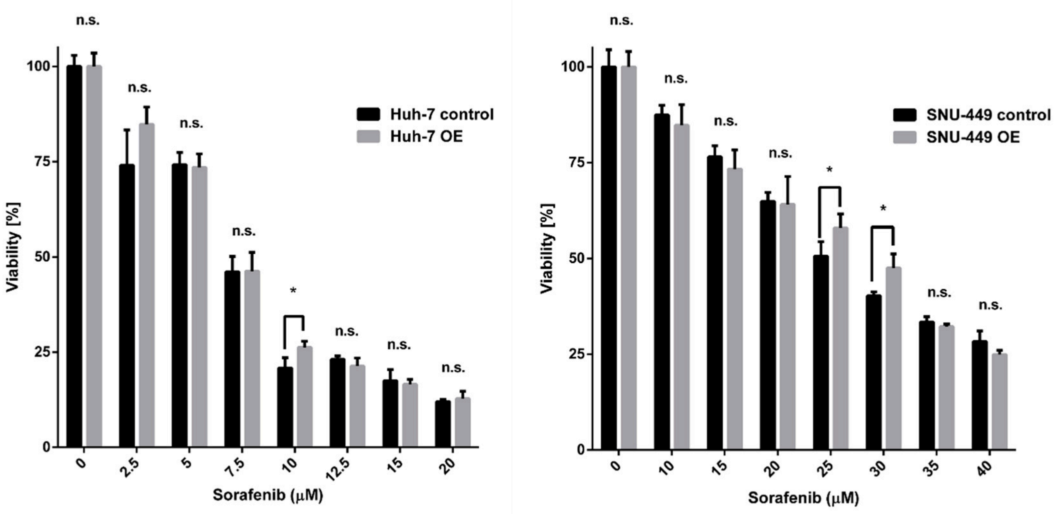

Figure S11: Viability of sorafenib treated OE cell lines measured by colorimetric CCK-8 assay (n = 4). Results are represented as averages and error bars represent standard deviation. * p-value ≤ 0.05; n.s. – not significant, Student`s t-test.

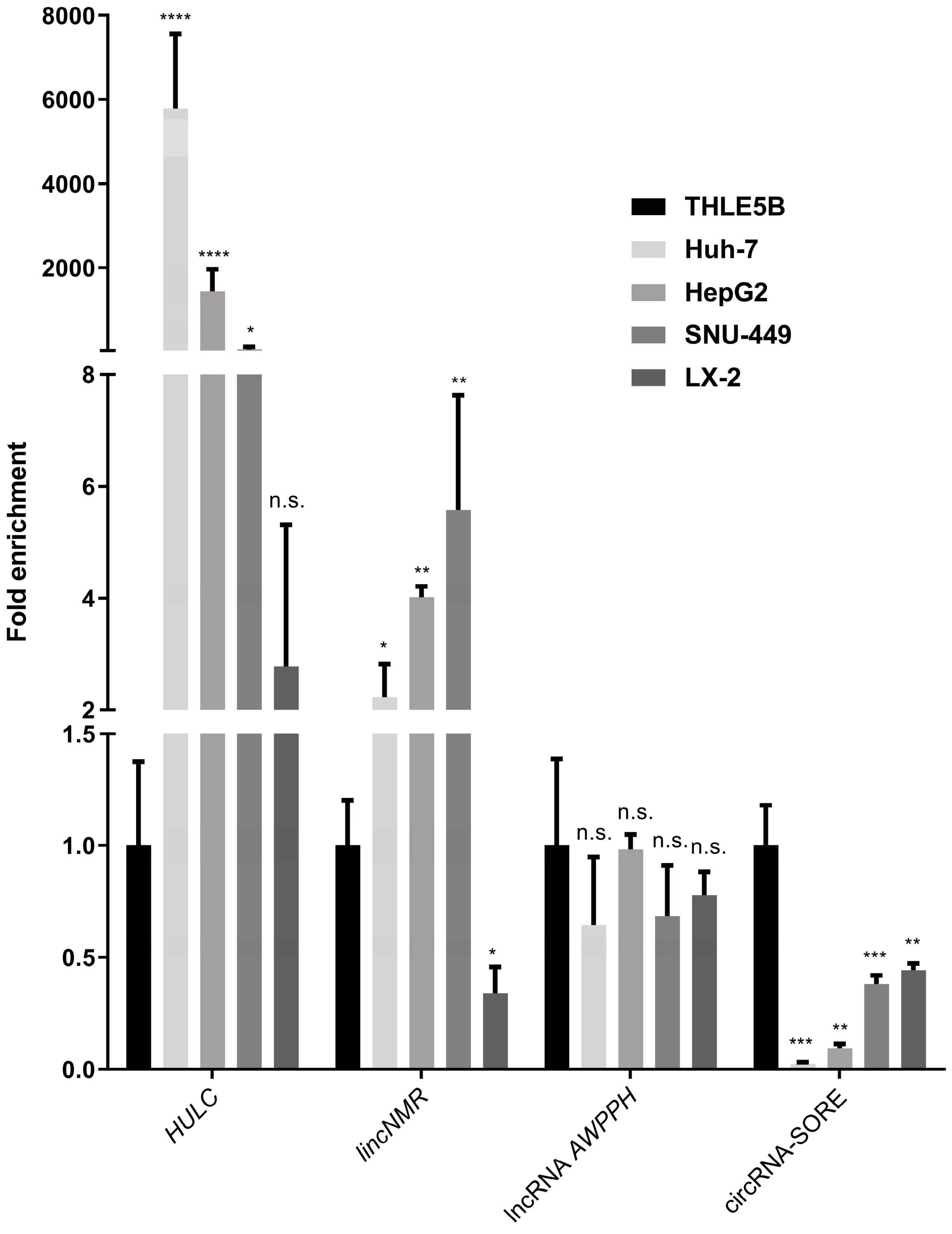

Figure S12: Relative expression of RNA species in human HCC model cell lines (Huh-7, HepG2, SNU-449) and human hepatic stellate cell line LX-2, normalized to the immortalized human hepatocyte cell line THLE5B (n = 3). Results are represented as averages and error bars represent standard deviation. * p-value ≤ 0.05; ** p-value ≤ 0.01; *** p-value ≤ 0.001; **** p-value ≤ 0.0001; n.s. – not significant, Student`s t-test.

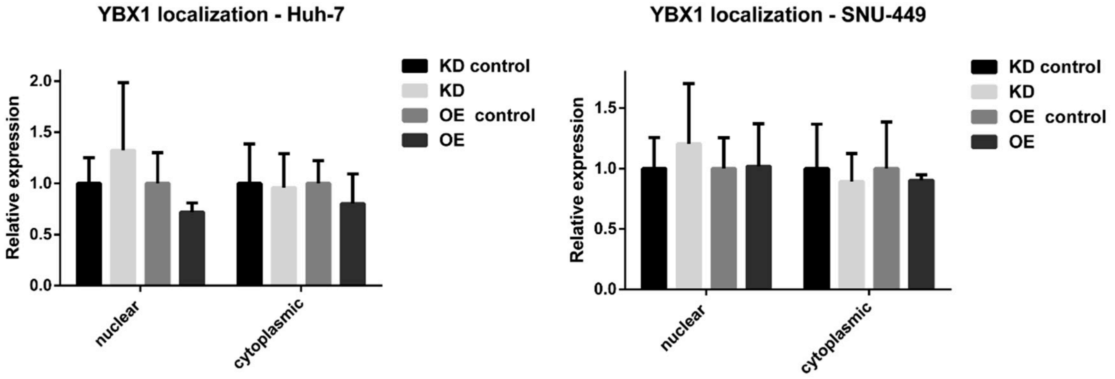

Figure S13: YBX1 localization in hsa_circ_0062682 stable cell lines identified by western blotting (n = 3); OE – overexpression, KD – knockdown, cyto – cytoplasmic fraction, nuc – nuclear fraction. Results are represented as averages and error bars represent standard deviation.
